# Machine learning and metagenomics reveal shared antimicrobial resistance profiles across multiple chicken farms and abattoirs in China

**DOI:** 10.1038/s43016-023-00814-w

**Published:** 2023-08-10

**Authors:** Michelle Baker, Xibin Zhang, Alexandre Maciel-Guerra, Yinping Dong, Wei Wang, Yujie Hu, David Renney, Yue Hu, Longhai Liu, Hui Li, Zhiqin Tong, Meimei Zhang, Yingzhi Geng, Li Zhao, Zhihui Hao, Nicola Senin, Junshi Chen, Zixin Peng, Fengqin Li, Tania Dottorini

**Affiliations:** 1 School of Veterinary Medicine and Science, University of Nottingham, Sutton Bonington, UK; 2 Shandong New Hope Liuhe Group Co. Ltd and Qingdao Key Laboratory of Animal Feed Safety, Qingdao, People’s Republic of China; 3 NHC Key Laboratory of Food Safety Risk Assessment, China National Center for Food Safety Risk Assessment, Beijing, People’s Republic of China; 4 Nimrod Veterinary Products Ltd., Moreton-in-Marsh, UK; 5 Shandong Kaijia Food Co., Weifang, People’s Republic of China; 6 Luoyang Center for Disease Control and Prevention, Luoyang City, People’s Republic of China; 7 Liaoning Provincial Center for Disease Control and Prevention, Shenyang City, People’s Republic of China; 8 Agricultural Biopharmaceutical Laboratory, College of Chemistry and Pharmaceutical Sciences, Qingdao Agricultural University, Qingdao City, People’s Republic of China; 9 Chinese Veterinary Medicine Innovation Center, College of Veterinary Medicine, China Agricultural University, Beijing City, People’s Republic of China; 10 Department of Engineering, University of Perugia, Perugia, Italy; 11 Centre for Smart Food Research, Nottingham Ningbo China Beacons of Excellence Research and Innovation Institute, University of Nottingham Ningbo China, Ningbo, People’s Republic of China

**Keywords:** Antimicrobial resistance, Bacterial genes, Machine learning, Bioinformatics, Metagenomics

## Abstract

China is the largest global consumer of antimicrobials and improving surveillance methods could help to reduce antimicrobial resistance (AMR) spread. Here we report the surveillance of ten large-scale chicken farms and four connected abattoirs in three Chinese provinces over 2.5 years. Using a data mining approach based on machine learning, we analysed 461 microbiomes from birds, carcasses and environments, identifying 145 potentially mobile antibiotic resistance genes (ARGs) shared between chickens and environments across all farms. A core set of 233 ARGs and 186 microbial species extracted from the chicken gut microbiome correlated with the AMR profiles of *Escherichia coli* colonizing the same gut, including *Arcobacter, Acinetobacter* and *Sphingobacterium*, clinically relevant for humans, and 38 clinically relevant ARGs. Temperature and humidity in the barns were also correlated with ARG presence. We reveal an intricate network of correlations between environments, microbial communities and AMR, suggesting multiple routes to improving AMR surveillance in livestock production.

## Abstract (web summary)

A data mining and machine learning approach for improving surveillance reveals clinically relevant antimicrobial resistance profiles across chicken farms, abattoirs and environments in three provinces in China.

## Main

Antimicrobial use in poultry production in China is five times higher than the international average^1^. Antibiotic use, even at low levels, alters and expands the gut resistome in livestock^2^, and the microbial community can shape antimicrobial resistance (AMR) phenotypes^3^. External events such as changes in diet, temperature and stress^4,5^ may result in the colonization of new resident species or AMR transfer between species^6^. Temperature, humidity and both bacterial species abundance and the presence of antibiotic resistance genes (ARGs)^7–9^ can influence bacterial infection in broilers^10^. Links between environmental conditions and AMR are particularly relevant for China and low- and middle-income countries (LMICs), where maintaining stable environmental conditions in industrial-scale farming may be challenging compared with in high-income countries^11^.

AMR surveillance in non-healthcare domains has not been widely adopted^12^, but is key to understanding how food production systems contribute to the selection and dissemination of antibiotic-resistant bacteria (ARB) and ARGs. Machine learning (ML) and big data mining offer tools to advance precision poultry farming^13,14^. Culture-based approaches involving whole genome sequencing (WGS) of individual pathogens, antibiotic susceptibility testing and ML techniques are effective predictors of genomic characteristics linked to AMR for both *Escherichia* *coli* isolates^15–18^ and other bacteria^19–24^. However, surveillance approaches focusing solely on WGS of individual pathogens may not capture the diversity of the microbial communities and resistomes within livestock production and ARG data may be missed^25^. In a recent proof-of-concept study, we observed that several ARGs present in the chicken faecal resistome were found to correlate with the resistance/susceptibility profiles of *E. coli* isolates cultured from the same samples^26^.

In this study, we developed a reference method for metagenomic-based surveillance targeting Chinese livestock farming, where AMR surveillance is particularly challenging, using an approach that takes into consideration the lack of laboratory resources commonly experienced in China and LMICs^27,28^. We used *E. coli* as an indicator species for AMR within the wider context of the microbial community populating the chicken gut. To address wider contexts, we explored the impacts on the microbiomes of the surrounding and connected farm environments, barn temperature and humidity, and adopted antimicrobial administration protocols.

## Results

### Birds and environment share clinically relevant mobile ARGs

**Fig. 1:**
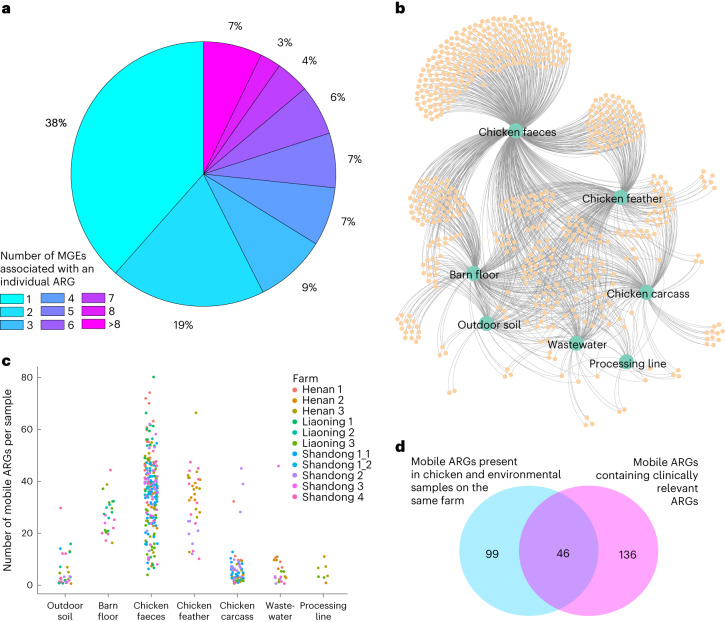
Analysis of potentially mobile ARGs. **a**, Pie chart showing the proportion of ARGs (out of the 195 found) associated with one or multiple MGEs. **b**, Undirected network graph showing potentially mobile ARGs (small orange circles) associated with different sample sources (large green circles). The edges in the graph link the potentially mobile ARGs to the sources in which they were found. **c**, Number of potentially mobile ARGs per sample per source. Each circle represents a single sample, with circles coloured by farm. **d**, Venn diagram showing that 145 (out of 661) potentially mobile ARGs were found to be present in both chicken and environmental samples from the same farm, and 182 potentially mobile ARGs contained clinically relevant ARGs. An overlap of 46 clinically relevant^30^ potentially mobile ARGs was found in chicken and environmental sources obtained from the same farm. Note that in this analysis, samples from the same source collected at *t*_1_ (week 3) and *t*_2_ (week 6) were aggregated together, leading to a total of seven sources considered for each farm.

Biological samples were collected from ten large-scale commercial poultry farms (see Methods, Supplementary Information, Supplementary Fig. 1 and Supplementary Tables 1 and 2). Microbial communities and ARGs were differentiated across farm sources and between farms and abattoir (Supplementary Information, Supplementary Figs. 2–5 and Supplementary Tables 3–5). As gene mobility may influence ARG presence across sources and because of the potential importance of mobile genetic elements (MGEs) in the development of effective surveillance systems^29^, we looked for ARGs that were within 5 kilobases (kb) of an MGE^26^ and considered these MGE–ARG combinations to be potentially mobile ARGs. In total, 661 different MGE–ARG combinations (potentially mobile ARGs) were found, featuring 195 unique ARGs (Supplementary Table 6). Of these, 75 ARGs (38%) were found in only one MGE–ARG combination, while the remaining 120 (62%) were found in multiple combinations (2 to 22; Fig. 1a). Over half (56%) of the 661 potentially mobile ARGs were present in more than one source (Fig. 1b), with three MGE–ARG combinations (IS*1216*-*poxtA*, IS*15*-*APH(3*′*)-Ia* and IS*Cfr1*-*AAC(3)-IId*) present in all sources except feathers. Chicken faeces had the highest number of potentially mobile ARGs, but also the greatest variance (Fig. 1c). Feathers and barn floor also carried many potentially mobile ARGs, the mean number statistically equivalent to faeces (Dunn’s test adjusted *P* > 0.05). Outdoor soil, carcasses, processing line and wastewater generally had lower numbers of potentially mobile ARG patterns per sample, with these numbers differing significantly (Dunn’s test adjusted *P* < 0.01) from faeces and feather, but not from each other. In total, across all 10 farms, 145 different MGE–ARG combinations were found in bird and environmental sources on the same farm, with some of these appearing on multiple farms. Of these, 46 contained clinically relevant ARGs^30^ (Fig. 1d). Notably, we found *bla*_NDM-5_ in chicken faeces, feathers and environmental barn floor samples. This gene is commonly found on the IncX3 plasmid, which can be disseminated among humans, animals, food and environment^31^, although we did not confirm plasmid presence in our short-read metagenomic sequencing (MGS) data. Another important clinically relevant gene, *qnrS1*, was found in chicken faeces, feather, environmental barn floor and wastewater samples. This plasmid-mediated quinolone resistance gene is known to be present in the chicken supply chain and is capable of being transferred to different bacteria^32^.

### *E. coli* AMR correlates with the gut microbiome it inhabits

We further investigated whether there was a correlation between the bacterial species found in the chicken gut, the resistome (that is, the ARGs from all species) and the AMR profiles of *E. coli* isolates taken from the same samples as the metagenome data. We cultured *E. coli* isolates from 170 chicken faecal samples (a subset of the samples that had been used for metagenomics) and characterized their AMR profiles against a panel of 26 antibiotics. The proportion of isolates resistant to each antibiotic ranged from 1% to 98% (Supplementary Table 7). All isolates were resistant to at least one antibiotic, with 169 resistant to at least three.

**Fig. 2:**
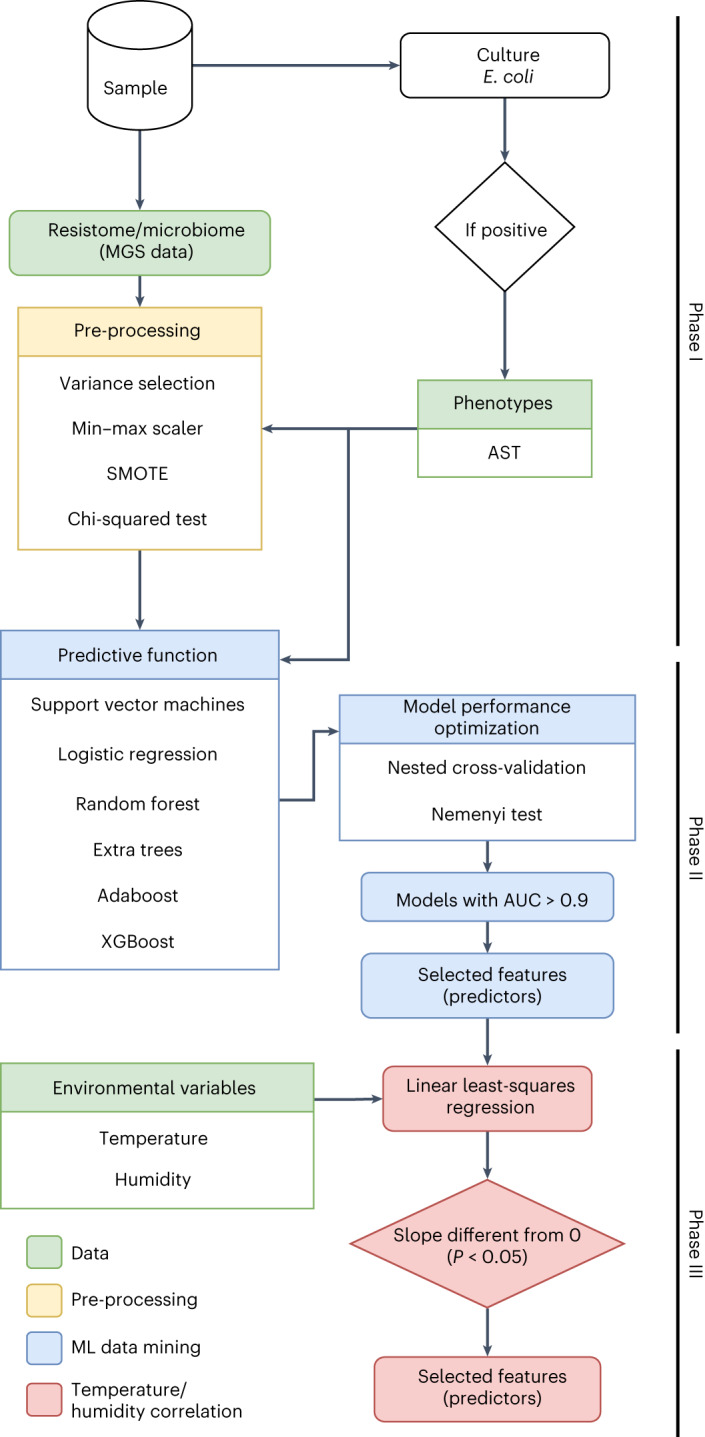
Data mining pipeline to find correlations between gut microbiome, antibiotic resistance in *E. coli*, temperature and humidity. The full data analysis workflow of the bespoke data mining method based on ML. Input data are shown in green. Phase I involves metagenome data pre-processing (in yellow). The steps are described in detail in the Methods section. Phase II involves the training and testing of ML-powered predictive functions to isolate metagenomic features (that is, the ARG count and relative abundances of microbial species present in the sample) correlated with phenotypic resistance (in blue). Phase III involves fitting regression models (discussed in the next section) to isolate metagenomic features that better correlate with variations of temperature and humidity (in red). AUC, area under the curve.

To investigate the correlations between antibiotic resistance in *E. coli* and gut microbiome, we developed a bespoke data mining method based on ML (Fig. 2). The method consists of building an ML-powered ‘predictive function’ whose input is the aggregation of information from the gut microbial community (relative abundances of microbial species) and gut resistome (ARG count) and whose output is the resistance of *E. coli* to a specific antibiotic (true or false) from antimicrobial susceptibility testing (AST). The predictive function was trained by using experimental data (supervised learning) and swapping different underlying ML technologies until optimal prediction performance was achieved. A set of the most informative features, also referred to as ‘predictors’, was extracted from the ML models. The set was then refined by analysis of the correlation with temperature and humidity (see later).

**Fig. 3:**
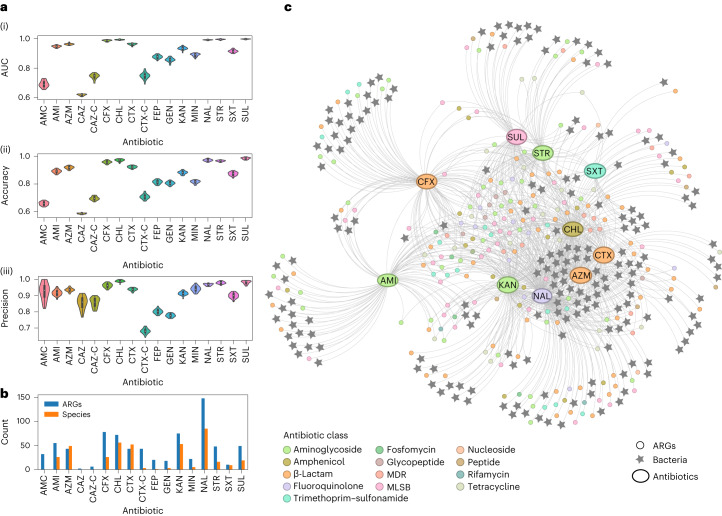
ML performance and feature selection from correlations between gut microbial species, resistome and antibiotic resistance in *E. coli*. **a**, Performance of the ML-powered predictive functions of *E. coli* resistance to specific antibiotics (ML technology: extra tree classifier; see Methods). Performance indicators (AUC, accuracy and precision) were computed as the average of 30 iterations of nested cross-validation (see Methods). See Supplementary Fig. 2 for performance indicator sensitivity, specificity and Cohen’s kappa score. The violin plots show the distribution of the data, with each data point representing one antibiotic model. Inside each violin plot is a box plot, with the box showing the interquartile range (IQR), the whiskers showing the rest of the distribution as a proportion of 1.5 x IQR and the white circle representing the median value. **b**, Counts of metagenomic features (ARGs and microbial species) found as the strongest predictors of *E. coli* resistance/susceptibility profiles to each antibiotic. **c**, Undirected graph showing the strongest predictors (metagenomic features in the chicken gut) for each antibiotic model. The edges of the graph link ARG or bacteria species nodes (predictor variables) to the antibiotic model in which they were found to be predictive. Both the ARG and antibiotic model nodes are colour coded according to the antibiotic class that the antibiotic/ARG is known to be associated with. The ML models were run for the following antibiotics: amoxicillin–clavulanic acid (AMC), amikacin (AMI), aztreonam (AZM), ceftazidime (CAZ), ceftazidime–clavulanic acid (CAZ-C), cefotaxime (CTX), cefotaxime–clavulanic acid (CTX-C), cefoxitin (CFX), chloramphenicol (CHL), cefepime (FEP), gentamycin (GEN), kanamycin (KAN), minocycline (MIN), nalidixic acid (NAL), streptomycin (STR), sulfafurazole (SUL) and trimethoprim–sulfamethoxazole (SXT). MDR, multidrug resistant.

Out of the 26 antibiotics, only 17 had sufficient data (resistance and susceptibility cases) to allow proper ML training: amikacin, amoxicillin–clavulanic acid, aztreonam, cefepime, cefoxitin, cefotaxime–clavulanic acid, ceftazidime, ceftazidime–clavulanic acid, chloramphenicol, cefotaxime, gentamycin, kanamycin, minocycline, nalidixic acid, streptomycin, sulfafurazole and trimethoprim–sulfamethoxazole. For all, the best prediction performance (Nemenyi test) was observed with the extra tree classifier (ML technology; Supplementary Table 8 and Supplementary Fig. 6). The prediction performance indicators computed using the extra tree method are reported in Fig. 3a and Supplementary Fig. 7. Ten predictive models (amikacin, aztreonam, cefoxitin, chloramphenicol, cefotaxime, kanamycin, nalidixic acid, streptomycin, sulfafurazole and trimethoprim–sulfamethoxazole) achieved performances exceeding AUC > 0.90.

Data mining showed that a core subset of the chicken gut resistome (all detectable ARGs from the chicken faeces metagenomic data) and microbial species (all bacterial species from the chicken faeces metagenomic data) exhibited strong predictive power for *E. coli* resistance. This core consisted of 419 features (186 microbial species and 233 ARGs) acting as strong predictors of *E. coli* resistance/susceptibility to 10 antibiotics (Fig. 3b,c and Supplementary Table 9) with an AUC of over 0.90. The 233 ARGs from the top 10 antibiotic models belonged to β-lactams (24% of the ARGs), aminoglycosides (18%), and macrolides, lincosamides and streptogramin B (MLSB; 18%), with other antibiotic classes accounting for less than 10% each. Of these 233 ARGs, based on the correlation of ARG read depth with species abundance (see Methods)^33^, 46 were found to be present in contigs identified as originating from *E. coli*. A further 16 ARGs (of the 233) were present only in contigs identified as other bacterial species (that is, they did not originate from *E. coli*). To further explore the relationship between core gut features and antibiotic resistance, the 419 features and 10 antibiotic resistances were visualized as nodes of a graph, with edges only connecting predictors to predicted resistances (Fig. 3c). This analysis highlighted a core of 66 ARGs (15 clinically relevant, including *bla*_NDM-5_, *bla*_CTX-M-15_, *dfrA15* and *dfra5*) acting as predictors of more than three antibiotic resistances. Three ARGs *(aphA6*, *vat(A)* and *vgb(A)*) were found to be predictors of eight antibiotic resistances. The same analysis revealed 28 microbial species in the gut acting as predictors of 5 antibiotic resistances (aztreonam, chloramphenicol, cefotaxime, kanamycin and nalidixic acid). These 28 species included the bacterial genera *Arcobacter*, *Acinetobacter* and *Sphingobacterium* in addition to other commensal bacteria.

Shapley additive explanation values were used to explain the AMR-related features selected by ML (Supplementary Fig. 8). The top ten most important features found to predict resistance for each antibiotic model indicated that 41% of the features had their presence positively associated with the prediction of resistant phenotypes, while 59% had their absence positively associated with the prediction of the resistant phenotype, most notably for the antibiotic models nalidixic acid and streptomycin. Conversely, eight of the top ten features in each model were negatively associated with the prediction of resistance. The chloramphenicol antibiotic model had the highest number of ARGs known to confer resistance to the same antibiotic class (phenicol; *optrA* (ref. ^34^), *lsaE* (ref. ^35^) and *mel* (ref. ^35^)) or known to facilitate resistance to it (*oqxA* (ref. ^36^)).

### Temperature and humidity shape the gut microbiome linked to AMR

For the top ten antibiotic models, we developed bespoke regression models using individual gut features as independent variables (one model per variable) and temperature or humidity as dependent variables to ascertain whether model fitting would highlight a correlation (see phase III in Fig. 2 and Methods). Temperature and humidity were measured in all farms except Liaoning 1 (LN1) over a full chicken production cycle (Supplementary Table 10 and Supplementary Fig. 9). Amongst the original 419 features, 130 ARGs and 48 microbial species correlated with humidity, whilst 39 ARGs and 20 microbial species correlated with temperature (Supplementary Fig. 10 and Supplementary Table 11). The correlation with humidity was on average stronger (higher *R*^2^ values in the regression analysis, Supplementary Fig. 10). Of the 130 ARGs correlated with humidity, 22% were MLSB, 18% were β-lactams, 17% were aminoglycosides and 11% were tetracyclines. Of the 39 ARGs correlated with temperature, 23% were β-lactams, 18% were MLSB, 15% were aminoglycosides and 13% were glycopeptides. Nineteen ARGs correlated with both temperature and humidity, four of them clinically relevant (*qnrA1*, *qnrS2*, *bla*_NDM-1_ and *catA8*). Four microbial species from the phyla Proteobacteria (*Helicobacter pullorum* and *Alcaligenes faecalis*), Firmicutes (*Bacillus cereus* group) and Bacteroidetes (*Bacteroides stercoris*) correlated with both temperature and humidity. One species from Tenericutes (*Mycoplasma yeatsii*) correlated with temperature only, while other species from Proteobacteria, Firmicutes, Bacteroidetes and Actinobacteria correlated with either temperature or humidity (Supplementary Table 11).

**Fig. 4:**
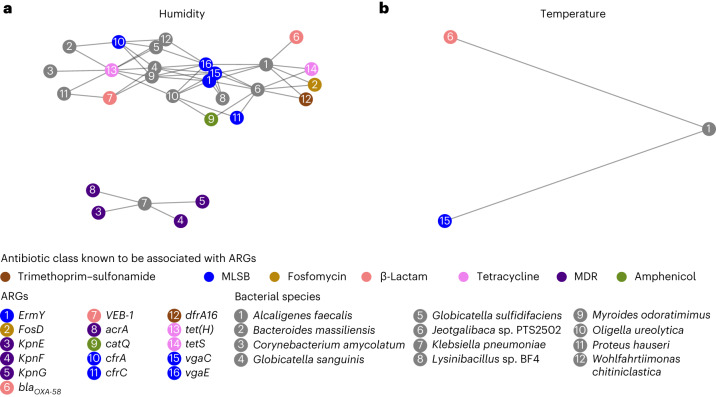
Gut features identified as predictors of *E. coli* resistance. **a**,**b**, Microbial species and ARGs correlated with humidity (**a**) and temperature (**b**). Microbial species and ARGs are correlated with humidity or temperature, and also with each other, indicating that the ARGs are likely to be present in the species. Features were considered correlated if the slopes of the linear regression lines were significantly different from zero (*P* < 0.05 using a two-sided *t*-test). Nodes indicate ARGs or microbial species; edges connect species to ARGs likely present in the species. ARG nodes are colour-coded according to the antibiotic class known to be associated with the ARG; microbial species nodes are shown in grey.

We tested for the possibility that some ARGs found to be correlated with temperature or humidity might belong to microbial species that are also correlated with temperature and humidity. This was done by correlating ARG read depth with microbial species read depth as proposed by Tong et al.^33^. The analysis highlighted two distinct subgraphs correlated with humidity (Fig. 4a) and one correlated with temperature (Fig. 4b). Notably, one of the subgraphs correlated with humidity contained *Klebsiella pneumoniae* and four related ARGs (*kpnE*, *kpnF*, *kpnG* and *acrA*). The subgraph containing *A. faecalis* and ARGs *vga(C)* and *bla*_OXA-58_ was found in both analyses (that is, it correlated with both temperature and humidity).

**Fig. 5:**
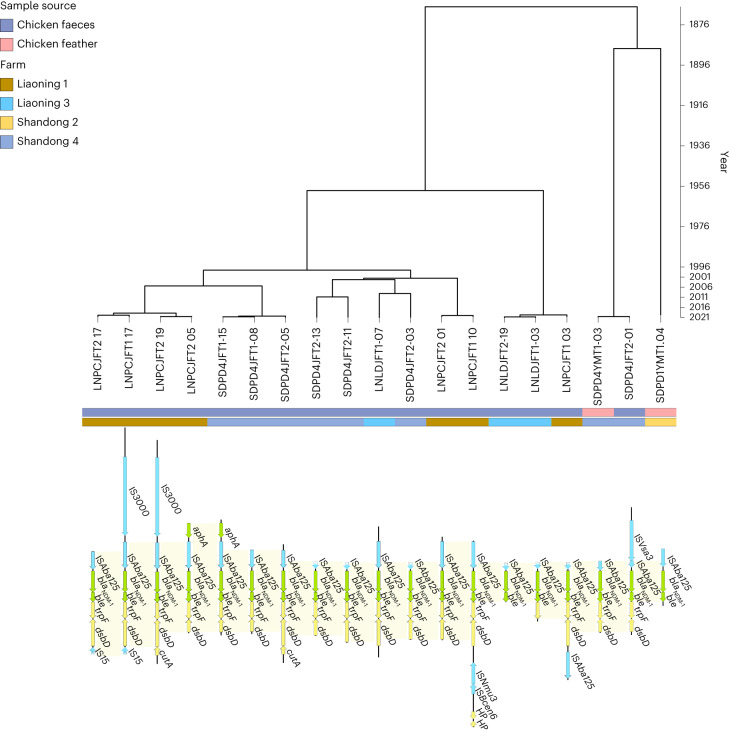
Gene structure and evolutionary analysis of the potentially mobile ARG pattern IS*Aba125*–*NDM-1*. Bayesian evolutionary phylogenetic tree reconstructing the phylogeny of contigs containing the clinically important ARG *bla*_NDM-1_ and MGE IS*Aba125*. Sample IDs (for example, LNPCJFT2-17) are given under the phylogenetic tree. The source type and location of the samples are indicated by coloured strips. The gene structure of each sample is shown below the tree with MGEs coloured blue, ARGs coloured green and other genes coloured yellow. The ARG *ble* is co-located with *bla*_NDM-1_ in all contigs.

We then investigated whether the gut ARG features identified as predictors of resistance in *E. coli*, and further identified as correlated with humidity or temperature, were in close proximity to MGEs. Ten ARGs were found located in close proximity to MGEs (MLSB: *optrA*, *mph(F)* and *erm(X)*; β-lactams: *bla*_NDM-1_ and *bla*_OXA-58_; amphenicol: *catA8* and *catB2*; aminoglycoside: *aadA1*; fluroquinolone: *qnrS2* and *qnrA1*). Three of the ten ARGs were found to be associated with only one MGE (*catB2* with IS*Pa25*, *mph(F)* with IS*15* and *qnrA1* with IS*15*), whilst the other seven were associated with two to nine different MGEs. All the MGE–ARG pairs were investigated for conserved structure across farms or sources. For example, the clinically important *bla*_NDM-1_ was found in close proximity to IS*15* in four samples (three chicken faeces from LN1 and one barn floor sample from Liaoning 3 (LN3); Supplementary Fig. 11). In 19 samples from chicken faeces and feather samples from LN1, LN3, Shandong 2 (SD2) and Shandong 4 (SD4), *bla*_NDM-1_ was found in proximity to MGE IS*Aba125* and located next to another ARG, *ble*, which is a known association for plasmid-borne *bla*_NDM-1_ in *Enterobacteriaceae* species from Asian regions^37^. Despite having found the same *bla*_NDM-1_–IS*Aba125* pattern in several farms (LN1, LN3, SD2 and SD4), there was no evidence of transmission between farms (Fig. 5). Instead, evolutionary analysis of the contigs (using a molecular clock model to predict the rate of molecular evolution on each branch of the phylogenetic tree^38^) suggested recent branching of isolates within individual farms (most common recent ancestor (MCRA) less than 2 years on most branches) and much earlier MRCAs between different farms (greater than 20 years), indicating the likelihood of this potentially mobile ARG widely circulating in livestock throughout China.

### The microbiome linked to AMR correlates with antibiotic use

We investigated whether the core chicken gut microbiome previously identified as predictors of resistance in *E. coli* may in turn be associated with antibiotic use on farms (measured by whether an antibiotic class was used or not used on the farm during the study period, Supplementary Table 12; see the Supplementary Information and Supplementary Fig 12 for additional details). We found that tetracycline, lincosamide or aminoglycoside use was associated with altered counts for 21 ARGs and 20 microbial species (Supplementary Information). We also found that 20 of the 21 ARGs for which the counts were significantly correlated with antibiotic use (indicated in Supplementary Fig. 13) were also correlated with humidity. Additionally, one of these genes, *erm(X)*, was also correlated with temperature. Of the 20 microbial species that were found to have a statistically significant difference in relative abundance in relation to antibiotic use (Supplementary Table 13 and Supplementary Fig. 14), 7 were correlated with changes in humidity (*Wohlfahrtiimonas chitiniclastica, Lachnoclostridium* sp. An76*, K. pneumoniae, Klebsiella variicola, Lysinibacillus* sp. BF 4*, Proteus hauseri* and *Proteus mirabilis*) whilst four were correlated with changes in temperature (*Alistipes* sp. An66, *Lactobacillus aviarius*, *Enterococcus cecorum* and *Enorma massiliensis*) with the *B. cereus* group correlated with both temperature and humidity.

## Discussion

Conventional AMR surveillance approaches fail to accurately assess bacterial and resistome diversity both within and between farms^39^. In our study, we used large-scale metagenomic sampling and *E. coli* isolation in combination with statistical and ML methods to draw out complex correlations showing AMR trends and patterns. *E. coli* has an established role as a reference indicator of AMR^26^. We found that 38 clinically relevant ARGs correlated with resistance to multiple antibiotics. Some of these antibiotics had no previously known association with these ARGs. In particular, 14 ARGs (*aadA16*, *aph(3*′*)-Ia*, *aph(3*′*)-VIa*, *bla*_CARB-16_, *catQ*, *dfrA15*, *dfrA16*, *dfrA27*, *bla*_OXA-58_, *bla*_PER-1_, *qnrD1*, *tet(Z)*, *tet(39)* and *bla*_SHV*-*110_) found to be associated with resistance to the highest number of antibiotics had previously been found in earlier studies on poultry in China^40–42^, confirming our method. However, we found a cluster of gut bacteria that correlated well with *E. coli* resistance to five different antibiotics. These included *Arcobacter* (an emerging waterborne and foodborne zoonotic pathogen, responsible for gastroenteritis in humans^43^), *Acinetobacter* (commensal in the poultry gut, but capable of causing extraintestinal diseases in both humans and poultry^44^) and *Sphingobacterium* (clinically relevant in humans and animals^45^). This result suggests that, in agreement with previous studies^12,29,46–50^, focusing exclusively on *E. coli* within the farm for surveillance purposes may not be as effective as monitoring a larger number of pathogens.

In our study, the farms that used tetracyclines, lincosamides and polypeptides were positively correlated with the presence of ARGs from a wide range of classes, beyond those specific to the selected antibiotics. This appears to be consistent with previous findings^51,52^, but contrasts with a recent study from the United States^53^. It is possible that the co-localization of AMR genes is playing an important role in AMR selection in our farms. Indeed, the co-localization of AMR genes in bacterial genomes in food animals has previously been observed and recognized as a food safety concern in China^54^ as well as elsewhere^55,56^.

The chickens in our study were housed in sheds that did not have an effective climate control system, and therefore experienced substantial temperature and humidity variations. Our results indicate that the core features of the gut microbial community and resistome, found to be correlated with resistance in *E. coli*, are also correlated with changes in temperature and humidity in chicken housing. Our results confirm and expand findings of previous studies^7–9,26,57^. Of note, the relative abundance of *A. faecalis* and the ARGs *vga(C)* and *bla*_OXA-58_ originating from this species (via analysis of ARG and species read depths) were found to be correlated with changes in both temperature and humidity. A greater abundance of *A. faecalis* and more severe clinical symptoms in higher humidity conditions have been observed previously in a case–control study of turkeys kept at different humidity levels and inoculated with *A. faecalis*^58^. This bacterium is commonly found in birds^59^ and would not typically be monitored by conventional surveillance. However, it is considered an emerging pathogen, has been associated with infections in humans and is considered difficult to treat due to its capacity to become extensively drug resistant^60^. Similarly, the important opportunistic pathogen *K. pneumoniae* and four ARGs (*kpnE*, *kpnF*, *kpnG* and *acrA*) originating from this bacterium and important for *K. pneumoniae* resistance^61^ were found to be correlated with changes in humidity. *K. pneumoniae* can be transmitted via airborne contamination and has previously been found to have increased survival in indoor high-humidity conditions, highlighting the importance of studying this bacterium in indoor environments^62^. The associations between environmental variables (easily monitorable and controllable) and the species and genes associated with AMR present opportunities for the development of novel AMR monitoring solutions, especially in LMICs where these variables are not controlled and pose a risk to the animals that are exposed to changes in them.

Ten potentially mobile ARGs in the gut resistome were found to correlate with *E. coli* resistance and with temperature and humidity. In addition, 67 potentially mobile ARGs were found to correlate with *E. coli* resistance and humidity. One of these, the gene *cfr(C)*, encoding a ribosomal RNA methyltransferase conferring resistance to linezolid and phenicol antibiotics, was found near IS*Ec9* (within 5 kb) and is associated with CTX-M genes^63,64^ (as we also found). The association of *drfA*16 with the transposase IS*6100* has previously been reported in only a single study with an association with *Corynebacterium diphtheriae*, the causative agent of cutaneous diphtheria^65^. These associations potentially indicate an environment-specific evolution of these MGEs, as has been hinted at in previous work on pig farms that showed the importance of MGEs for AMR varied according to the season^66^.

Even though our analysis relied on a large set of samples from many heterogeneous sources with geographical and seasonal variations, our scope was limited to *E. coli*, did not consider human samples and would benefit from extending the analysis to other indicator species such as *Enterococcus*^67^. Spatial and temporal variations in farm/slaughterhouse microbial communities and resistomes are mirrored in human faecal samples, as our previous work and that of others have shown^26,68^, but whether these observations would be generalizable and globally true is currently unknown.

Metagenomic sequencing has the potential to broaden our knowledge of the factors driving resistance and improve AMR surveillance^69^. Metagenomic sequencing data are essential for developing new infection and resistance control policies, raising awareness of AMR and allowing the optimal use of antibiotics by veterinary professionals^12,70^. MGS shows great promise for AMR surveillance in environmental sectors, but methodologies need to be standardized and data gaps filled^71,72^, with few laboratories and countries at present having both the resources and expertise to use MGS for AMR surveillance^73^. With further development, metagenomic and ML approaches could be deployed to provide fast and reliable predictions of AMR outbreaks, emerging pathogens and transmission routes^69^.

Despite the increasing availability of low-cost precision farming technologies and metagenomics^29,74^, innovation and methodological advances must further enable the development of surveillance solutions capable of monitoring AMR dynamics^12,29^. Drug resistance arises from complex interactions across ARBs, microbial communities, geographical niches and environments, evolutionary forces, climate and human practices. We have demonstrated how methodologies can be developed that are capable of associating a wide array of microbial species and genes with observable AMR, and further assessed how those are associated with the environmental variables of temperature and humidity. Consideration of all relevant and interconnected AMR datasets in a 360° approach will drive forward our understanding and control of AMR spread.

## Methods

### Ethics statement

This study complied with all relevant ethical regulations and was specifically performed in accordance with protocols approved by the Ethics Committee of the State Key Laboratory of the China National Center for Food Safety Risk Assessment (ethical approval number: 2018018). Ethical approval was also obtained from the Research Ethics Committee of the School of Veterinary Medicine and Science at the University of Nottingham (application identification number: 2340 180613).

### Collection of biological samples and environmental sensor data

For this study, we selected ten large-scale commercial poultry farms in three different provinces in China (Shandong, Henan and Liaoning; hereafter, the farms are denoted SD*x*, HN*x* and LN*x*, respectively), covering an area of 472,500 km^2^, each farm feeding into one of four regional abattoirs (two in Henan, one in Liaoning and one in Shandong). Each farm featured multiple barns, each barn containing between 12,000 and 32,800 birds, leading to a total production capacity of 110,730 to 380,000 birds per breeding cycle (depending on farm). Broiler production was based on self-breeding with broilers bred on the farm and moved to barns in same-aged batches. Of the ten selected farms, four (three in Liaoning and one in Shandong) used net housing systems, whilst the other six used cage housing systems. During collection, the number of birds per barn did not significantly differ between the two housing systems (*t*-test, *P* = 0.07).

Sampling followed the same pooled birds over one breeding cycle, except for one farm in the Shandong province (Shandong 1), which was sampled over two cycles to conduct a pilot study to fine-tune the collection campaign and data analysis protocols^26^. Biological samples were collected at the same three time points in every breeding cycle (including both cycles in Shandong 1): *t*_1_ (week 3), *t*_2_ (week 6) and *t*_3_ (1–5 days after week 6). Biological samples consisted of pooled faeces and feathers (not necessarily from the same animals) from the droppings of live birds in the barns collected from the barn floor immediately after excretion at mid-life (*t*_1_) and at the end of life (*t*_2_) of the animals, as well as barn floor samples (litter) collected at the same time points. In the abattoirs, samples were collected on slaughtering day (*t*_3_) from carcasses, meat processing surfaces (referred to as the processing line) and wastewater. Soil samples were collected from outside areas surrounding the farms at *t*_1_ and *t*_2_. Details of the collection methods are available in the Supplementary Information.

All the farms involved in this study were equipped with heating/air conditioning systems. Environmental sensor data (temperature and humidity) were collected at intervals of 5 min using the automated sensors and data loggers available in most farms (HN1, HN2, HN3, SD2, SD3 and SD4). Three farms (SD1, LN2 and LN3) were unequipped with automated solutions and manual measurements were performed using SMART SENSOR AS837 temperature/humidity devices either daily or every 6 h. Farm LN1 had technical issues with the sensor and did not acquire any measurements. In all cases, the temperature and humidity data were averaged over three measurements taken at different locations within the barn.

### DNA library construction and sequencing

DNA extraction was performed on faeces, barn floor and outdoor soil samples using a magnetic bead genomic DNA extraction kit (DOP336-T3, TIANGEN Biotech). For carcass samples, the cetyltrimethylammonium bromide method^75^ was used. Samples with DNA content above 1 µg were used to construct the DNA library. The DNA concentration was measured using a Qubit dsDNA Assay Kit and Qubit 2.0 fluorometer (LifeTechnologies), and the integrity was measured using 1% agarose gel electrophoresis. A total amount of 1 μg DNA per sample was used as input material for the DNA sample preparations. Sequencing libraries were generated using NEBNext Ultra DNA Library Prep Kit for Illumina (NEB). The DNA sample was fragmented into 350 bp, and then DNA fragments were end-polished, A-tailed and ligated with the full-length adaptor for Illumina sequencing with further PCR analysis. Finally, the PCR products were purified (AMPureXPsystem) and the libraries analysed for size distribution using an Agilent2100 Bioanalyzer (Agilent Technologies) and quantified using real-time PCR. After cluster generation, the library preparations were sequenced on an Illumina Novaseq 6000 platform and 150 bp paired-end reads were produced.

### Bioinformatics analysis

The raw sequence reads, obtained from the Illumina HiSeq sequencing platform, were pre-processed and filtered using Readfq (V8, https://github.com/cjfields/readfq) to acquire high-quality data for subsequent analysis. Host DNA was filtered using Bowtie 2 (v2.3.4.1)^76^ and SAMtools (v1.9 (ref. ^77^); reference genome accession code: GCF_000002315.6). The microbiomes of samples were constructed by assembling the metagenome sequencing data for the different sample sources (chicken faeces, chicken feather, chicken carcass, barn floor, outdoor soil, wastewater and processing line) separately using binning and dereplication pipelines^26,78^. MEGAHIT (v1.1.2)^79^ software was used to assemble the sequences. Single sample assemblies were generated for all samples with MEGAHIT default parameters. Co-assemblies were generated for each sample source group (chicken faeces, chicken feather, chicken carcass, barn floor, outdoor soil, wastewater and processing line), each with the MEGAHIT setting parameters “--continue --kmin-1pass --min-contig-len 1000” as previously used for co-assemblies^78^. Filtered contigs (>2,000 bp) were mapped to single assemblies and co-assemblies using Burrows–Wheeler Aligner–Maximal Exact Match (BWA-MEM v2-2.1)^80^ and SAMtools (v1.9)^77^ to produce the Binary Alignment Map (BAM) files. METABAT2 (v2.15)^81^ was used obtain the depth of coverage. The taxonomic classification and composition (relative species abundances) of the metagenome reads were profiled using MetaPhlAn (v3.0)^82^ with Bowtie 2 (v2.3.4.1)^76^ using the default settings –bowtie2out–input_type fastq. Nonmetric multidimensional scaling (NMDS) of the relative species abundance was performed in R (v3.6.2) using the vegan^83^ package with Bray–Curtis dissimilarity. Analysis of variance was performed in R using PERMANOVA from the vegan package^83^ with pairwise testing using the pairwise adonis function^84^ with Holm correction for multiple comparisons. Relative abundances were visually analysed by combining violin plots and categorical scatter plots, and differences were assessed by Wilcoxon rank sum test with Holm correction (adjusted *P* = 0.05).

As sequencing depth can affect the observed diversity in genomic sequencing, rarefaction is widely used to normalize samples before analysis across different sample types^85^. However, the use of rarefaction is controversial as the subsampling leads to the loss of information available in the non-rarefied sample^86^. Hence, in this study, we used rarefied data only where necessary (to compare different sample types) and used non-rarefied data where only a single sample type was being considered. Host-removed reads were rarefied using the minimum sample depth using seqtk (https://github.com/lh3/seqtk), with the random seed fixed for each pair of reads.

### Analysis of resistome and MGEs

Assembled genomes were searched for sequence similarity to annotated ARGs present in the Comprehensive Antibiotic Resistance Database (CARD)^61^ using Basic Local Alignment Search Tool–nucleotide (BLASTn)^87^ with an identity threshold of 95% and coverage threshold of 95% (stricter thresholds with respect to our previous study^26^) to minimize the likelihood of mislabelled ARG variants. NMDS analysis was performed on the resulting gene count matrix in the vegan R package^83^ using Bray–Curtis dissimilarity. Comparisons were made using (1) the total number of ARGs present per sample, (2) the actual count of individual ARGs per sample and (3) the relative ARG abundance per antibiotic class according to CARD (the number of ARGs present in the sample divided by the total number of ARGs in that class). These three approaches were visually analysed by combining violin plots and categorical scatter plots, and differences were assessed by Wilcoxon rank sum test with Holm correction (adjusted *P* = 0.05).

To identify the source bacteria from which the ARGs originated, in accord with a previous study^33^, rarefied reads from each metagenome sample were mapped to their single assemblies using BWA-MEM (v2-2.1)^80^ and SAMtools (v1.9)^77^. The average depths were assigned to the ARG-carrying contigs and ARGs. The coverage of ARGs, normalized by gene/contig length^33^, was then used to correlate with species abundance through the Spearman correlation test. ARG–species pairs were considered significantly correlated if *P* < 0.05 and the Pearson correlation coefficient ≥ 0.6.

To look for the presence of potentially mobile ARGs shared across different sources, ARGs carried by both the environment and chickens were considered. Filtered contigs (>500 bp) in each assembly were searched for ARGs and MGEs by a BLASTn search against the CARD^61^ and ISfinder (https://isfinder.biotoul.fr/) databases using an identity threshold of 95% and coverage threshold of 95% to prevent false positives and variant uncertainty^88^. The distance between an ARG and MGE was calculated from the positions of the ARG and MGE in the contig^26^. ARG-carrying contigs with a distance of more than 5 kb between ARG and MGE were discarded^68,89–91^, with the remaining contigs classed as potentially mobile ARGs. Contigs were annotated using Prokka (v1.14.6)^92^. Potentially mobile ARG patterns found in only a single sample were discounted in the analysis. ARGs were further classified as clinically important if the ARG was included in the Risk I category (clinically important ARGs dataset) according to Zhang et al.^30^. These genes were classed as Risk I if they were (1) present in human-associated environments, (2) potentially mobile genes and (3) present in ESKAPE pathogens (*Enterococcus faecium*, *Staphylococcus aureus*, *Klebsiella* *pneumoniae*, *Acinetobacter baumannii*, *Pseudomonas aeruginosa* and *Enterobacter* species). The structures of the potentially mobile ARG patterns (MGE type, ARG carried, MGE carried, sample source, farm, number of samples carrying potentially mobile ARG and distance) are summarized in Supplementary Table 8. For IS*Aba125*–*bla*_NDM-1_, the gene structure was visualized using EasyFig^93^.

Evolutionary phylogeny was reconstructed for contigs carrying the potentially mobile ARG IS*Aba125*–*NDM-1* using BEAST (v1.10.4)^38^. All combinations of three clock models (strict, uncorrelated log normal and uncorrelated exponential) and three tree priors (constant coalescent, logistic growth and Bayesian skyline) were tested using stepping-stone sampling on the contigs to identify the best model. The best model was found to be a random uncorrelated log-normal clock model with a Bayesian skyline growth model. The GTR-gamma nucleotide substitution model was used, as selected by a maximum likelihood tree analysis in IQ-tree2 (v2.0.6) using automated model selection^94^. The analysis was conducted for three independent chains until the effective sample size, that is, the effective number of independent draws from the posterior distribution, for all parameters was greater than 200 per chain. This entailed each chain running for 100 million steps. Convergence was assessed in Tracer (v1.7.1)^95^, and chains were subsequently combined using LogCombiner (v1.10.4)^96^. The maximum clade credibility tree was selected using TreeAnnotator (v1.10.4) and then visualized in iTOL (v5)^97^.

### Investigation of correlations between faecal metagenomic features, antibacterial resistance and temperature/humidity

*E. coli* strains were taken from the same samples as the chicken gut metagenome data and cultured and used as indicator species for AMR^98^ for each chicken faeces sample (see Supplementary Information for details of the culture and AST methodology). Only 191 of the 223 samples were positive for an *E. coli* isolate. Of these 191 samples, a further 21 (from LN1) were discarded from this analysis as technical issues with the environmental sensors resulted in these samples not having the necessary temperature and humidity data needed for the ML pipeline. Therefore, 170 samples remained to be analysed by the ML pipeline.

The antibiotic susceptibility/resistance profiles of the *E. coli* strains were evaluated against a panel of 26 antibiotics (Supplementary Table 1) using broth microdilution and interpreted according to the criteria of the Clinical and Laboratory Standards Institute^99^. The overall data analysis pipeline, implemented in Python (v3.9.15)^100^ and SciPy (v1.9.3)^101^ consisted of three phases (Fig. 2):

- Phase I: pre-selection of metagenomic features. For each antibiotic, isolation of a first set of faecal metagenome features (that is, ARG counts and relative abundance of microbial species) showing correlation with the resistance/susceptibility profiles of *E. coli* based on a chi-squared test.
- Phase II: assessment of the feature predicting power through the development of ML-powered predictive functions. Development of ML-based predictive functions of resistance/susceptibility (one predictive function per antibiotic) that operate from the pre-selected features (see below for more details), supervised training with available samples and then inspection of the best-fit state of each predictive function to retrieve the predictive influence of each feature, that is, the relative weight of the feature in driving the prediction result.
- Phase III: assessment of feature dependency on temperature/humidity through the development of ML-powered regressors. Development of ML-based regressors to identify correlations between the set of faecal metagenome features identified in phase II and temperature/humidity conditions.

The three phases are described in detail below.

### Phase I

An initial set of features was considered for each of the 26 antibiotics and comprised all data on ARG count and microbial species abundance in the faecal metagenome. The following steps were applied to process and reduce such sets using the Python package Scikit-learn^102^:

1. Abundances were turned into relative abundances (0–1 range) using min–max normalization.
2. For each specific antibiotic, imbalances in sample size between resistance and susceptibility observations were compensated with synthetically generated data using the synthetic minority oversampling technique (SMOTE)^103^, adopting five-nearest neighbours as the default parameter.
3. Features (ARG count and relative abundance of species) with a variance equal to zero (that is, features that had the same value in all samples) were removed as redundant (incapable of acting as effective predictors).
4. Features that did not show strong association with the prediction result (resistance/susceptibility profile), according to a chi-squared test, were removed (all the features with a *P* value greater than 0.01 were removed). No multiple comparison correction was used as we were looking to assess each feature in its own right^104^.
5. The remaining set of features were subjected to visual inspection via a graph representation designed to create spatial clusters that highlight correlation. The analysis was performed using the NetworkX^105^ library in python. In the resulting graph, nodes representing features (ARG count or relative abundance of species) are connected to nodes representing resistance/susceptibility to a specific antibiotic if the existence of a correlation had been demonstrated by the chi-squared test (see the previous step). The nodes were spatially arranged using the Kamada–Kawai path-length cost function^106^.

### Phase II

Predictive functions based on multiple underlying ML technologies were developed and tested, each trained to predict resistance/susceptibility to a specific antibiotic, using the features pre-selected in Phase I as input of supervised learning. A predictive function was trained and validated for each of the 26 antibiotics tested. Upon successful training and validation, inspection of the best-fit state of each predictive function allowed retrieval of the quantitative influence of each feature (that is, relative weight) in relation to predicting resistance/susceptibility to each antibiotic.

The following ML technologies were tested for implementation of the predictive functions: logistic regression, linear support vector machine, radial basis function support vector machine, extra tree classifier, random forest, Adaboost and XGBoost, all implemented using the Python package Scikit-learn^102^. Nested cross-validation (NCV)^107^ was used to assess the performance and select the optimal hyperparameters for each technology. NCV is an iterative procedure in which different configurations of the predictive function (that is, different hyperparameters driving the selected technology) are repeatedly tested for performance whilst reshuffling the training and testing sets. NCV consists of an outer loop dedicated to randomly reallocating observations into new training and testing sets, and an inner loop where different configurations (sets of hyperparameters) for the predictive function are tested with the current training and testing set. In our analysis, we ran an NCV with a fivefold outer loop (five reshuffles of the training and testing sets) and a threefold inner loop (three reshuffles of the training set) for each different ML technology. Prediction performance was measured via the receiver operating characteristic area under the curve (ROC-AUC, referred to simply as AUC in the following), accuracy, sensitivity, specificity and precision, all computed at each iteration of the outer loop^108^. Thirty iterations of the NCV assessments were completed for each ML technology. The technologies were then compared by running an *F*-test on the mean quantitative results for each using the AUC metric. A minimum of 12 samples in the minority class were required for the classification for SMOTE and NCV. Nine antibiotics (ampicillin, ampicillin–sulbactam, cefazolin, ciprofloxacin, doxycycline, imipenem, levofloxacin, meropenem and tetracycline) lacked sufficient samples in one class to allow cross-validation and SMOTE, and so were not taken further. We compared the seven ML architectures to avoid bias in the analysis related to choosing a specific ML technology. Prediction performance was measured using 30 NCV iterations, with the final performance score defined as the mean of all runs. The Nemenyi test was used to verify which predictive function performed best out of the seven ML methods. The extra tree predictive functions ranked best according to all studied performance indicators apart from sensitivity (where all the predictive functions were considered statistically equivalent) and were finally selected to produce the correlation results. As the extra tree method had been selected to power the final predictive functions, Gini importance was used to extract the strongest predictors from the final, trained models.

### Phase III

The last phase of the analysis consisted of the development of regression models to identify correlations between the set of faecal metagenome features identified in Phase II (predictors) and temperature/humidity conditions. Only the predictors extracted from ML-powered models with AUC > 0.9 were considered.

A separate regression model was created to represent the relationship of each predictor (considered as the input/explanatory variable) with either temperature or humidity (considered as the dependent variable). The predictor was treated as continuous if related to either a relative microbial abundance or ARG count. Temperature and humidity values were collected at each farm and averaged from the 7 days before the two time points *t*_1_ and *t*_2._

Each regression model was developed using linear least-squares fitting (using the Python package SciPy^101^) using the coefficient of determination (*r*^2^) to assess the goodness of fit. Metagenome features were considered to be significantly correlated with temperature or humidity if the slope of the regression line statistically differed from zero (*P* < 0.05 using the Wald test with *t*-distribution of the test statistic). We looked for correlations between the ARG read depth and species read depth, which would indicate the likelihood of ARGs originating from a particular species, as proposed by Tong et al.^33^. An undirected graph was created using NetworkX (v2.8.4)^105^ to visualize the interconnected ARGs and species selected by the regression framework for humidity and temperature.

### Analysis of antibiotic use bias

The correlations observed between the metagenomic data in chicken faeces and the resistance profiles observed in *E. coli* may be influenced by the different antibiotic protocols that each farm adopted (Supplementary Table 13). To identify whether the differences in antibiotic treatment in each farm led to bias in the selected metagenomic features, we calculated the relative abundance of ARGs expressed by first grouping ARGs by relationship to each specific antibiotic, and then by computing ratios of ARGs present in the sample, divided by the total number of ARGs for each antibiotic, and then calculated the relative abundance of the microbial species. For these three cases, we used the Wilcoxon rank sum test to verify whether there was a difference between the samples from farms that received an antibiotic against the samples that did not receive that antibiotic.

### Statistical analysis

For details of all statistical analyses, see the Supplementary Information.

### Reporting summary

Further information on research design is available in the Nature Portfolio Reporting Summary linked to this article.

## Supplementary information

Supplementary information Supplementary research objectives, results, methods, Figs. 1–14 and list of tables.

Reporting Summary

Supplementary Tables Supplementary Tables 1–13.

## Data Availability

The metagenomic sequencing data supporting the conclusions of this article are available in the NCBI database under Bioproject accession numbers PRJNA678871 (for Shandong 1_1 and 1_2) and PRJNA841806 (for all other farms). In addition, the reference genome used for filtering host DNA is available in the NBCI database under accession code GCF_000002315.6. All source data needed to recreate the figures are provided in the Supplementary Data 1.

## Code availability

The code is available via Github at https://github.com/tan0101/Commercial_MGS2023. The code was used for the machine learning classification, regression analysis and network analysis.
