# Supplementary material for: Machine learning and metagenomics reveal shared antimicrobial resistance profiles across multiple chicken farms and abattoirs in China
Source: Nat Food. 2023 Aug 10;4(8):707–20. doi: 10.1038/s43016-023-00814-w (PMC10444626; doi:10.1038/s43016-023-00814-w)
Supplement: Supplementary file 1 — Supplementary research objectives, results, methods, Figs. 1–14 and list of tables. [file 43016_2023_814_MOESM1_ESM.pdf]

nature food

Article

https://doi.org/10.1038/s43016-023-00814-w

Machine learning and metagenomics reveal shared antimicrobial resistance profiles across multiple chicken farms and abattoirs in China

In the format provided by the authors and unedited

Machine learning and metagenomics reveal shared antimicrobial resistance profiles across multiple chicken farms and abattoirs in China

Contents:

Supplementary Research objectives and Background

Supplementary Results

Supplementary Methods

Supplementary Figures 1-14

List of Supplementary Tables

Supplementary References

Supplementary Research Objectives and Background

The structure of the work described here, as well as the objectives that motivated it, are summarised in the following.

i. We wanted to gain insight into AMR trends in the Chinese poultry industry. To do this, we conducted a comprehensive longitudinal study across three provinces, spanning 2.5 years. Our primary objective was to collect metagenomics samples from chickens, carcasses, and their breeding and slaughtering environments in ten large-scale commercial poultry farms and connected abattoirs. Additionally, we sought to employ multi-scale analysis techniques to investigate the similarities, differences and spread of the resistome, microbiota and MGEs within and across the studied ecological contexts;

ii. We wanted to search for correlations between resistance in the E. coli colonizing the gut (detected using AST) and the entire resistome and composition of the microbial community within the same gut, assessed by MGS. We also wanted to explore how variations in resistome and composition of the microbial community would correlate to the susceptibility of E. coli to different antibiotics;

iii. We wanted to investigate the correlations between temperature and humidity fluctuations within the barn, and variations of resistome and composition of the microbial community in the gut as detected by MGS, focusing in particular on the subset of variations previously found correlated with resistance in E. coli;

iv. In performing the above analyses, we also wanted to develop and present our bespoke analysis method as a general-purpose tool for exploring correlations amongst phenotypic manifestations, MGS data, and external variables (in this case temperature and humidity), applicable beyond the specific subject illustrated here, and putatively superior or at least complementary to conventional analysis of MGS data, capable of providing a significant support to the development of surveillance solutions for AMR.

From more effective solutions for surveillance, a better understanding of the mechanisms involved in AMR in livestock may arise. This understanding may in turn lead to the possible identification of a larger number of options in terms of what production and environmental variables should be kept under observation when monitoring for infection and AMR in livestock. Importantly, this may lead to reducing the need for culture-based analysis, a key strategic milestone in particular for LMICs, due to lack of lab resources and the cost of on-field analysis.

An element of distinction, with respect to the state of the art, is the way metagenomics is used within our method. The conventional approach to metagenomics is to run comparisons between the MGS data and existing databases to identify ARGs and associated mobile genetic elements (MGEs)[1]. However, with the conventional approach, our knowledge on the putative functional role of the found genetic elements remains limited to what has been annotated in the databases. On the contrary, many more functional interactions between ARGs and other relevant genetic traits and microbial species may be present and continuously changing in livestock, given the evolving nature of the microbiomes in the chicken gut and connected environments.

We have recently demonstrated[2] that, instead of limiting the analysis of MGS data to retrieving known matches between genetic elements and functional annotations within existing databases, we can encode information extracted from metagenomic data into a feature vector, and then search for statistical correlations between such feature vector and selected phenotypical manifestations (e.g. infection, development of a resistance trait to some antibiotic, etc.).

Machine learning in particular can be used to identify elements of the feature vector most strongly correlated to observed phenotypical variation, which allows to identify additional potential involvement of genetic elements in phenotype-related mechanisms, beyond what currently annotated. Correlation alone does not necessarily demonstrate causal dependency. Nevertheless, the method allows to isolate interesting candidates for further investigation.

Whilst recently there have been studies combining both metagenomic and culture-based analyses[3-6], none of these have attempted to perform a comprehensive analysis to find correlations between metagenomic data and phenotypic manifestations. Two recent studies used metagenomic samples to predict AMR and virulence determinants (by comparison of known genes in public databases) of clinical infections; however, typically these samples were mono-or polymicrobial with at most two species[7,8].

Supplementary Results

Sample collection campaign

Sample collection was performed on farms located in the three Chinese provinces of Shandong, Henan and Liaoning (Supplementary Figure 1, Supplementary Tables 1 and 2, further information in the Methods section). Sample collection resulted in a total of 461 viable biological samples, covering two time points in the bird life cycle within the farm (t1 and t2) and one time point in the slaughterhouse (t3). Biological samples consisted of bird

faeces (n = 223; 116 at t1, 107 at t2), feathers (n = 36; 17 at t1, 19 at t2), barn floors (n = 23; 10 at t1, 13 at t2), carcasses (n = 94 at t3), abattoir wastewater (n = 21 at t3), abattoir processing lines (n = 12 at t3), and outdoor soil (n = 52; 25 at t1, 27 at t2).

Bacteria communities and resistomes vary across farm sources

Taxonomic profiling of the metagenomic samples revealed 19 bacterial phyla, 2 archaea phyla and 3 eukaryote phyla (Supplementary Fig. 2A and Supplementary Table 3). The microbial communities formed two clusters (Supplementary Fig. 2B) (PERMANOVA, $p < 0.001$), clearly separating farm sources (faeces, feathers, barn floor and outdoor soil) from abattoir sources (carcasses, processing line and wastewater). Pairwise testing indicated that abattoir sources were not significantly separated from each other. However, farm sources were consistently separated from abattoir sources and from each other (adjusted p values < 0.05). Comparison of individual phyla abundances (Wilcoxon Rank-sum test) highlighted nine phyla with differences across sources (Supplementary Fig. 3 and Supplementary Table 4). As expected, typical soil phyla, Actinobacteria and Planctomycetes were more abundant in outdoor soil samples, and commensal gut species (Firmicutes, Protobacteria, and Bacteroidetes) were found in high abundances in most sample sources indicative of potentially contaminated environments. Chlamydiae, a phylum endemic in birds, was found in high abundance in abattoir samples.

In the resistome of all samples, after rarefying to the minimum read depth, 260 ARGs were found, representative of 14 antibiotic classes. The ARG count patterns differed in all farm and abattoir sources (PERMANOVA, $p < 0.05$) except for wastewater and abattoir processing line, Supplementary Fig. 4. The analysis of pattern differentiation for ARGs belonging to each specific class (Wilcoxon Rank-sum test, Supplementary Fig. 5 and Supplementary Table 5) revealed that 13 of the 14 classes had significant differentiation at least in one pairwise comparison across sources, consistent across farms. Specifically, barn floor (t1 and t2) samples carried a greater number of aminoglycosides, amphenicol, macrolide-lincosamide-streptogramin B (MLSB) and tetracycline genes compared to wastewater, outdoor soil, carcasses and (for t2 only) processing line (adjusted p values < 0.05). Chicken faeces carried a greater number of aminoglycoside, MLSB and amphenicol genes compared to outdoor soil, wastewater, processing line and carcasses (adjusted p values < 0.05), and beta lactam genes compared to outdoor soil, wastewater and carcasses (adjusted p values < 0.05). Additionally, chicken faeces collected at t1 carried a greater number of

multi-drug resistant genes (MDR) and fosfomycin genes compared to outdoor soil, wastewater and carcasses (adjusted p values < 0.05).

The microbiome linked to AMR correlates to antibiotic usage

We investigated if the core chicken gut microbiome previously identified as predictors of resistance in *E. coli* (i.e., bacterial species and ARGs found in the ML to be correlated to resistant-susceptibility phenotypes for the antibiotic models with an AUC greater than 0.90), may in turn be associated with antibiotics usage on farms (measured by whether an antibiotic class was used or not used on the farm during the study period, Supplementary Table 12). We found statistically significant differences (measured by a Wilcoxon rank sum test) in the relative abundance of ARGs per antibiotic class (Supplementary Fig. 12), counts of ARGs (Supplementary Fig. 13) and relative abundances of microbial species (Supplementary Fig. 14), between farms using and not using antibiotics (Supplementary Table 13). On the farms that received tetracycline antibiotics, the tetracycline class and tetracycline ARGs (*tet(39), tet(B),* and *tet(Y)*) were found to be significantly increased (adjusted *p* values < 0.05). In addition, in these farms there was also a significantly increased presence of genes from the classes: aminoglycoside, beta lactam, MLSB, MDR, phenicol, trimethoprim, fosfomycin, fluroquinolone, glycopeptide and nucleoside (adjusted *p* values < 0.05). All these, except fosfomycin, glycopeptide and nucleoside, had a greater than expected co-presence on contigs with tetracycline genes (Chi-square test, Holm correction adjusted *p* values < 0.0001). Similarly, on farms that received lincosamide antibiotics there was an increased presence of MLSB the gene *erm(42)*. There was also an increased presence of genes in aminoglycoside, beta lactam, phenicol and MLSB classes. All had a greater than expected co-presence on contigs with MLSB genes (Chi square test, Holm correction adjusted *p* values < 0.0001). For the farms that received aminoglycoside there was a decrease in the counts of the gene *ErmX* and an increase in the presence of genes from the classes: nucleoside and trimethoprim. For the farms that received polypeptide there was an increase in the counts of the genes *ErmC*, *MCR-1*, *mphE* and *spd* and an increase in the presence of genes from the classes: aminoglycoside, beta lactam, MLSB and tetracycline.

Supplementary Methods

Sample collection methods

For faecal samples, each sample consisted of approximately 10 g fresh sample of mixed chicken faeces (2-3 chickens), collected from the bottom of the chicken cage/net using a sterilized spoon. Feather samples were collected from the birds and swabbed using cotton tipped swabs. Pooled carcass samples were collected in the abattoirs using a sponge swab (SS100NB, Hygiena International, Watford, UK) on the surface of the carcass. In addition, samples from four types of environmental sources (barn floor, soil outside the barn, wastewater and processing line in the abattoir) were also collected. Barn floor samples were taken using a sterilized spoon. Wastewater samples of no less than 20 mL were collected from the water pipe or by using pipettes. Abattoir processing line samples were collected from multiple surfaces, e.g., the cutting table and transfer belt of the cutting and deboning house. Soil samples consisted of about 10 g soil, collected outdoors at depth of 1-3cm, 5m from the external barn walls, to ensure sufficient separation from areas of human use. All biological samples were collected using aseptic techniques, and then stored in secure containers at 4℃ during transportation to the laboratory and extracted within 24h.

Antibiotic susceptibility testing of *E. coli* isolates

For each sample, *E. coli* strains were cultured as indicator organisms. 1g sample of faeces and outdoor soil was vortexed with 9 mL of sterile buffered peptone water tube (BPW; Luqiao Inc., Beijing, China) for 1 min. Broiler carcass sponge samples were homogenised with 10 mL BPW for 1 min in a stomacher bag. Approximately 1 mL was added to 9 mL *E. coli* (EC) broth (Luqiao Inc.) and incubated at 37℃ for 16-20 h. A loopful of these solutions was then streaked onto an eosin-methylene blue (EMB) agar and MacConkey (MAC) Agar (Luqiao Inc.) and incubated at 37°C for 18-24 h. Typical *E. coli* colonies were counted and subsequently characterized by Bruker MALDI Biotyper (Germany).

The antimicrobial susceptibility testing was carried out on the cultured *E. coli* isolates. Antimicrobial susceptibility to a panel of agents was determined by broth microdilution and interpreted according to the criteria based on the Clinical & Laboratory Standards Institute (CLSI) interpretive criteria (CLSI 2009). The minimum inhibitory concentrations (MIC) of 28 antimicrobial compounds were measured for the *E. coli* isolates: ampicillin (AMP), ampicillin/sulbactam (AMS), tetracycline (TET), chloramphenicol (CHL), trimethoprim/sulfamethoxazole (SXT), cephazolin (CFZ), cefotaxime (CTX), ceftazidime (CAZ), cefoxitin (CFX), gentamicin (GEN), imipenem (IMI), nalidixic acid (NAL),

sulfisoxazole (SUL), ciprofloxacin (CIP), amoxicillin/clavulanic acid (AMC), cefotaxime/clavulanic acid (CTX-C), ceftazidime/clavulanic acid (CAZ-C), polymyxin E (CT), polymyxin B (PB), minocycline (MIN), amikacin (AMI), aztreonam (AZM), cefepime (FEP), meropenem (MEM), levofloxacin (LEV), doxycycline (DOX), kanamycin (KAN), streptomycin (STR). The resistance/susceptibility profiles for each isolate were calculated (summarised in Supplementary Table 1). *E. coli* ATCC™25922 was used as a control bacterium for these experiments.

Statistical Analysis

Analysis of variance was done using PERMANOVA in R, with pairwise testing based on the adonis function with Holm correction for multiple comparisons. Statistical comparison for the mean number of potentially mobile ARGs was done in R, using the Kruskal Wallis test with pairwise comparisons using a two-sided Dunns test with Holm correction. Additional statistical comparisons were made using the SciPy package implementing: 1. For the ARGs analysis a two-sided Wilcoxon Rank-sum test, with Holm correction - adjusted p value 0.05 was used to compare the number of ARGs present per sample, the counts of individual ARGs and the relative ARG abundance per antibiotic class; 2. For the taxonomic composition a two-sided Wilcoxon Rank-sum test, with Holm correction - adjusted p value 0.05 was used to compare the relative abundances of the phyla; and 3. a two-sided Friedman Statistical F-test (FF) with Iman-Davenport correction for statistical comparison of multiple datasets over the seven different classifiers used (p value 0.05). With 7 classifiers and 17 antibiotic models, the Friedman test is distributed according to the F distribution with $7-1 = 6$ and $(7-1)\times(17-1) = 96$ degrees of freedom. The critical value of $F(6,96)$ for p value = 0.05 is 2.19451621. The post-hoc Nemenyi test was used to find if there is a single classifier or a group of classifiers that performs statistically better in terms of their average rank after the FF test has rejected the null hypothesis that the performance of the comparisons on the individual classifiers over the different datasets is similar. For the correlation analysis with temperature and humidity, a linear least-square regression analysis was used and a ARG or a microbial specie was found statistically correlated to the temperature or humidity if the slope of the regression line statistically differed from 0 (p value < 0.05 using a two-sided t-test).

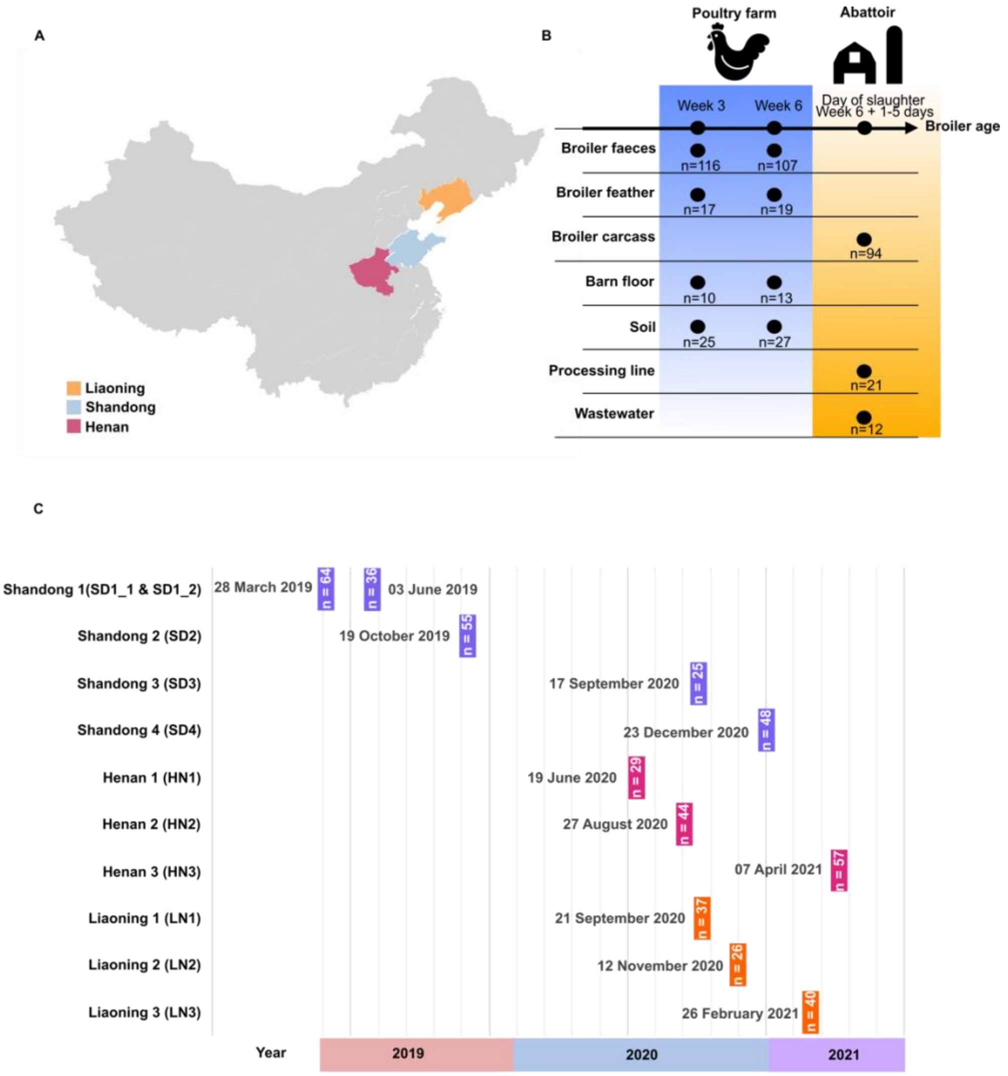

Supplementary Fig. 1: Summary of the collection of biological samples and environmental sensor data: (A) Map of mainland China[9] showing the three provinces of China (Liaoning, Shandong and Henan) where the 10 farms are located. Three farms were located in Liaoning province, four farms were located in Shandong province and three farms in Henan province. (B) Source and collection time points for the biological samples collected within each farm. Biological samples consist of: i) farm samples (faeces and feather from chickens and barn floor and outdoor soil samples) collected at mid-life (t_1: week 3) and at the end of life (t_2: week 6) and ii) abattoir samples (chicken carcass, processing line and wastewater) collected on slaughtering day (t_3: 1-5 days after week 6). The number of samples collected from each source type and collection timepoint is given; (C) Start dates of the collection campaign for each farm. Note the use of an underscore to distinguish the two collection campaigns executed to cover two breeding cycles at Shandong 1 (SD1_1 and SD1_2), which were sampled as part of a pilot study to optimise sampling and data analysis protocols.

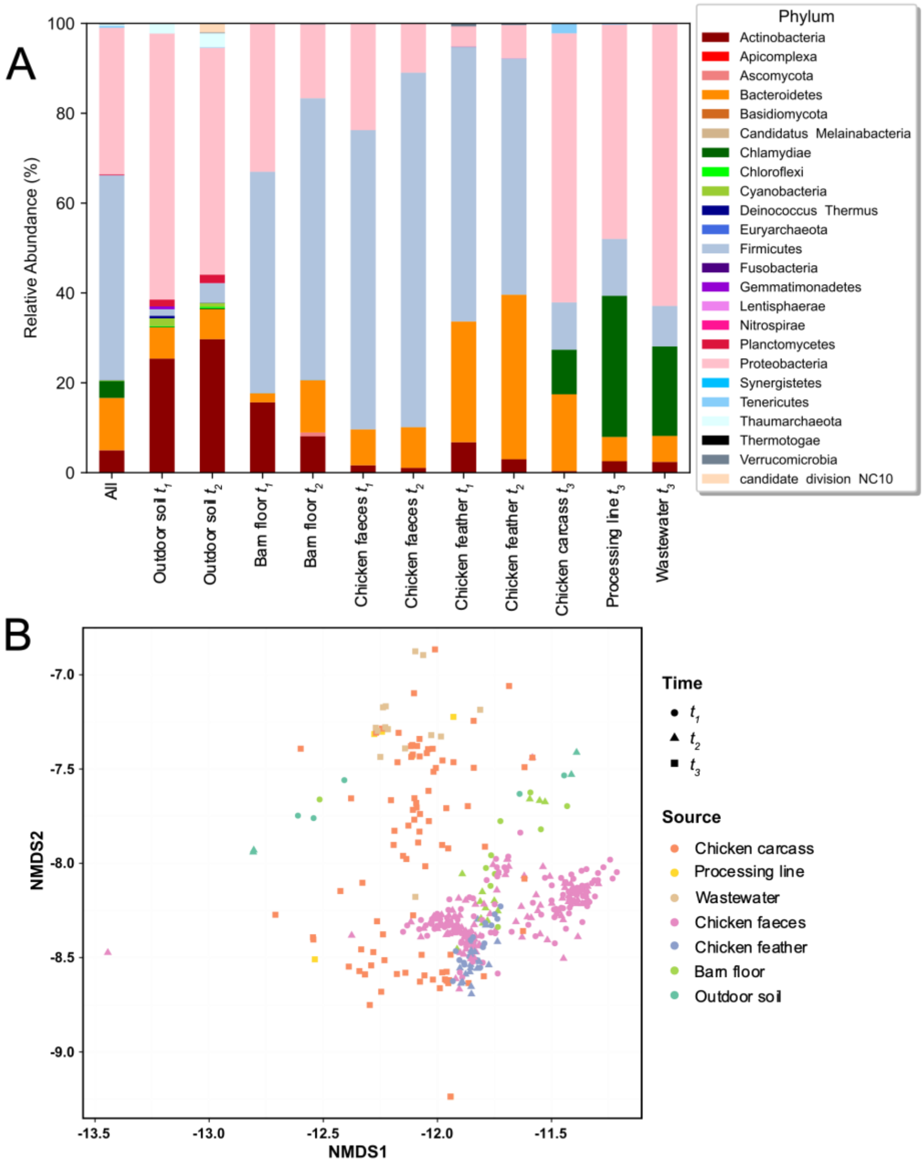

Supplementary Fig. 2. Abundance and diversity of the microbial community structure. (A) Taxonomy at phylum level of the metagenomes grouped by source (chicken carcasses, chicken faeces, chicken feathers, wastewater, processing line, barn floor and outdoor soil). (B) NMDS analysis based on Bray-Curtis dissimilarity of phylum relative abundances as calculated using MetaPhlan. The NMDS analysis depicts significant separation by source. Sample points are coloured according to source: outdoor soil (turquoise), carcasses (orange) feathers (violet), faeces (pink), barn floor (green) processing line (yellow) and wastewater (beige). The collection time point is given by shape: *t1* (circle), *t2* (triangle) and *t3* (square).

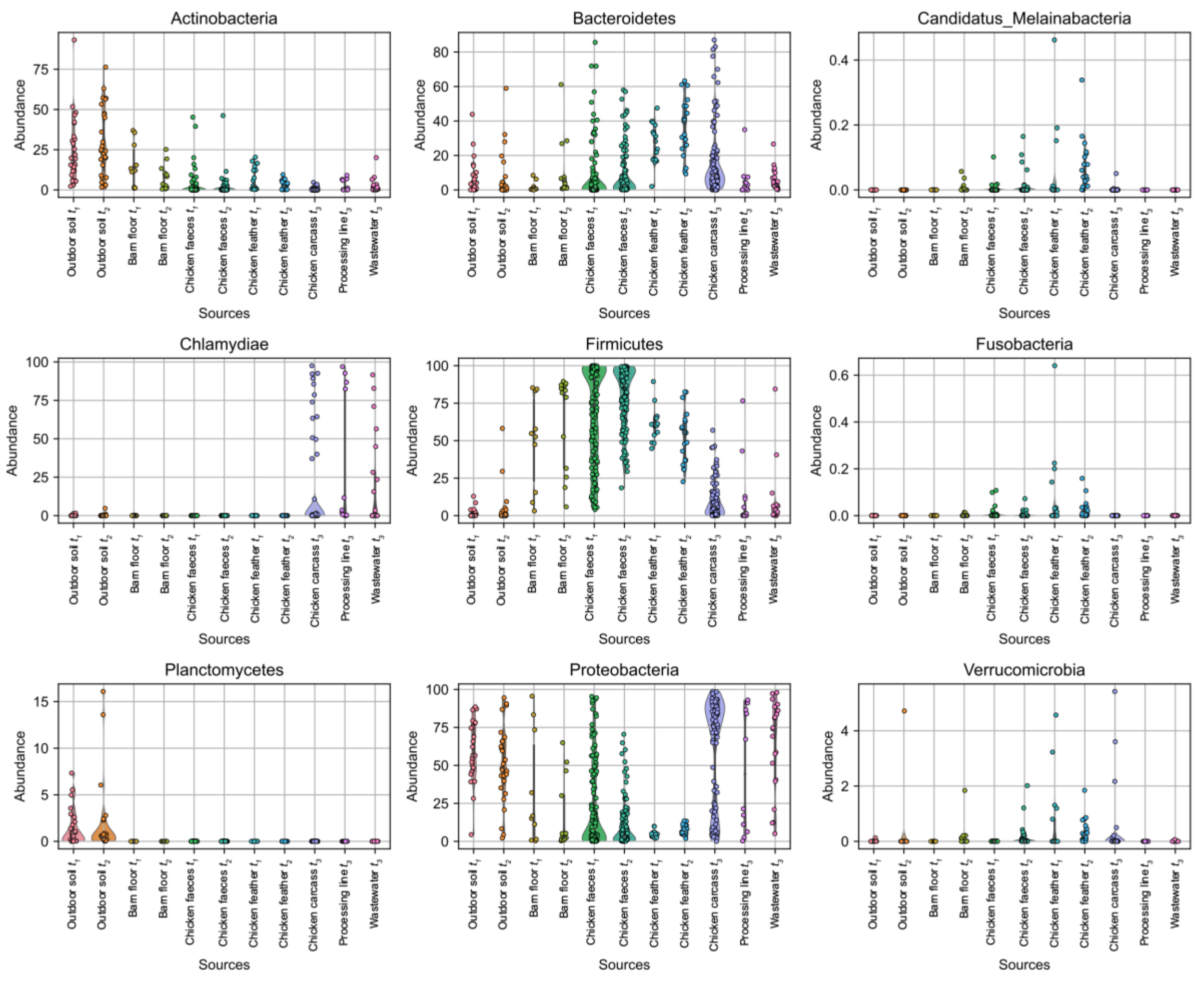

Supplementary Fig. 3. Relative abundance of nine phyla separated by sample source. Combined violin plot and categorical scatter plots showing the relative abundances of nine phyla which were found to have at least one case of statistically significant differentiation between sources. Each circle represents an individual sample.

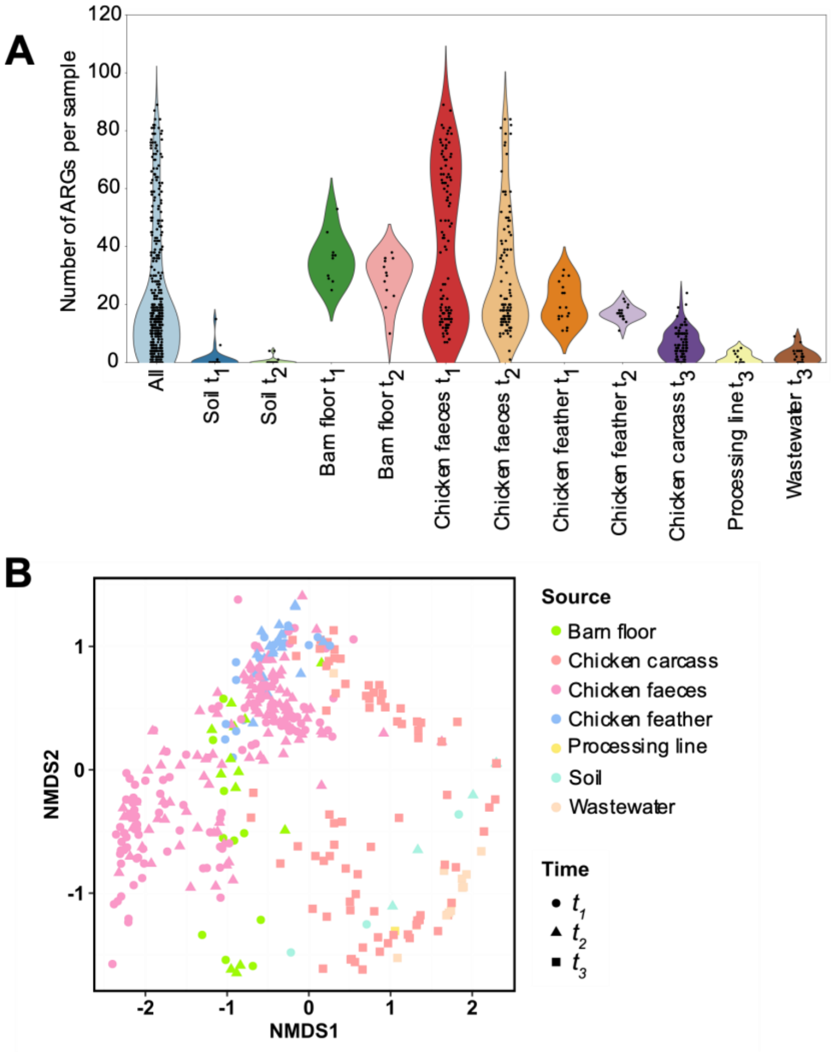

Supplementary Fig. 4. Occurrence of ARGs in environment, livestock and carcass samples in different sources and timepoints. (A) Abundance of the resistance genes in outdoor soil (t_1 and t_2), chicken carcass (t_3), chicken feather (t_1 and t_2), chicken faeces (t_1 and t_2), barn floor (t_1 and t_2), processing line (t_3) and wastewater (t_3) samples across all ten farms. (B) NMDS analysis of the ARGs of rarefied reads in chicken carcasses (orange), chicken faeces (pink), chicken feather (purple), outdoor soil (green), barn floor (light green), Abattoir processing line (yellow) and wastewater (brown) based on Bray-Curtis dissimilarity at time points t_1 (circles), t_2 (triangle), t_3 (squares).

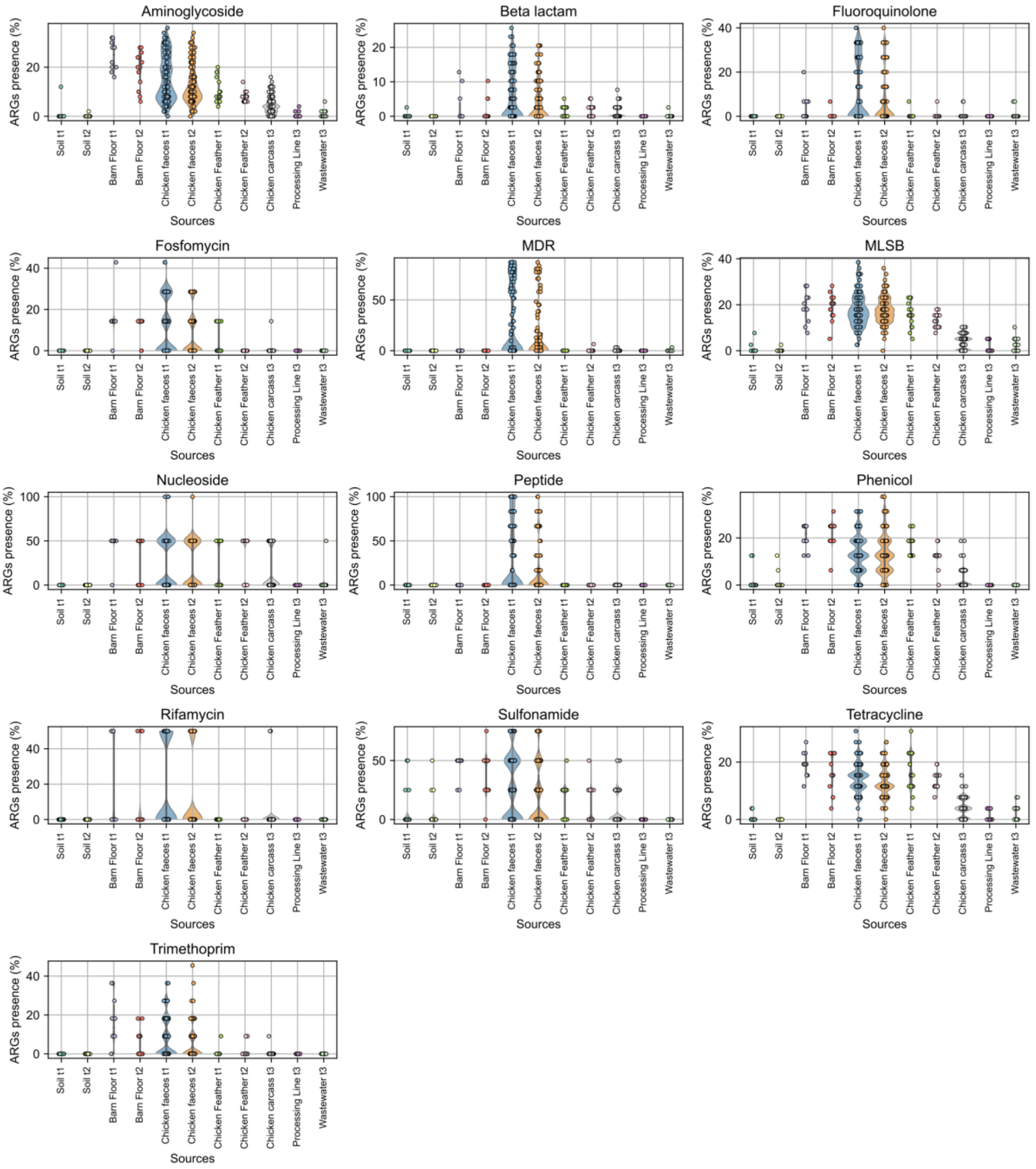

Supplementary Fig. 5. Relative presence of ARG classes across sources**.** Combined violin and categorical scatter plot for the 13 (out of 14) antibiotic classes that resulted in at least one case of significant differentiation between each other when compared across any two sources. Relative presence expressed as percentage (ARGs in sample / Total number of ARGs in the respective antibiotic class x 100). Each circle represents an individual sample.

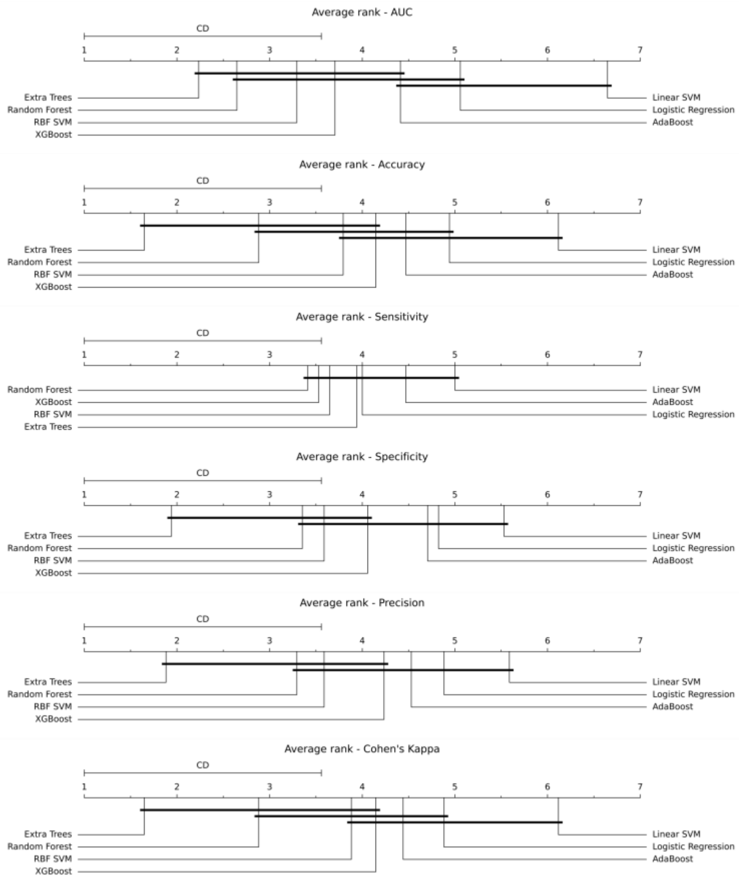

Supplementary Fig. 6. Nemenyi *post-hoc* test. Comparison of the performance of the 7 predictive functions, using their average ordinal rank over the 17 antibiotics analysed based on the performance metrics: (A) AUC, (B) accuracy, (C) sensitivity, (D) specificity, (E) precision and (F) Cohen's kappa score. The x-axis indicates the average ordinal rank of the machine learning methods. The scale is from 1 (best rank) to 7 (worst rank). For each performance metric, the ordinal rank of a predictive function is defined as: the ML method with the best performance is given rank 1, the second-best performance rank 2 and the *n*-th performance rank *n*, with *n* being the number of machine learning methods used. For each antibiotic and performance metric, the methods are ranked between 1 (highest performance) and 7 (lowest performance), since in this case there are 7 machine learning methods used. Next, for each method, the ranks are averaged based on the 17 antibiotics studied. The critical distance (CD) is defined based on the Nemenyi two-sided post-hoc test (p value = 0.01), all the methods that fall in the same bold bar below the axis are considered statistically equivalent based on the CD value.

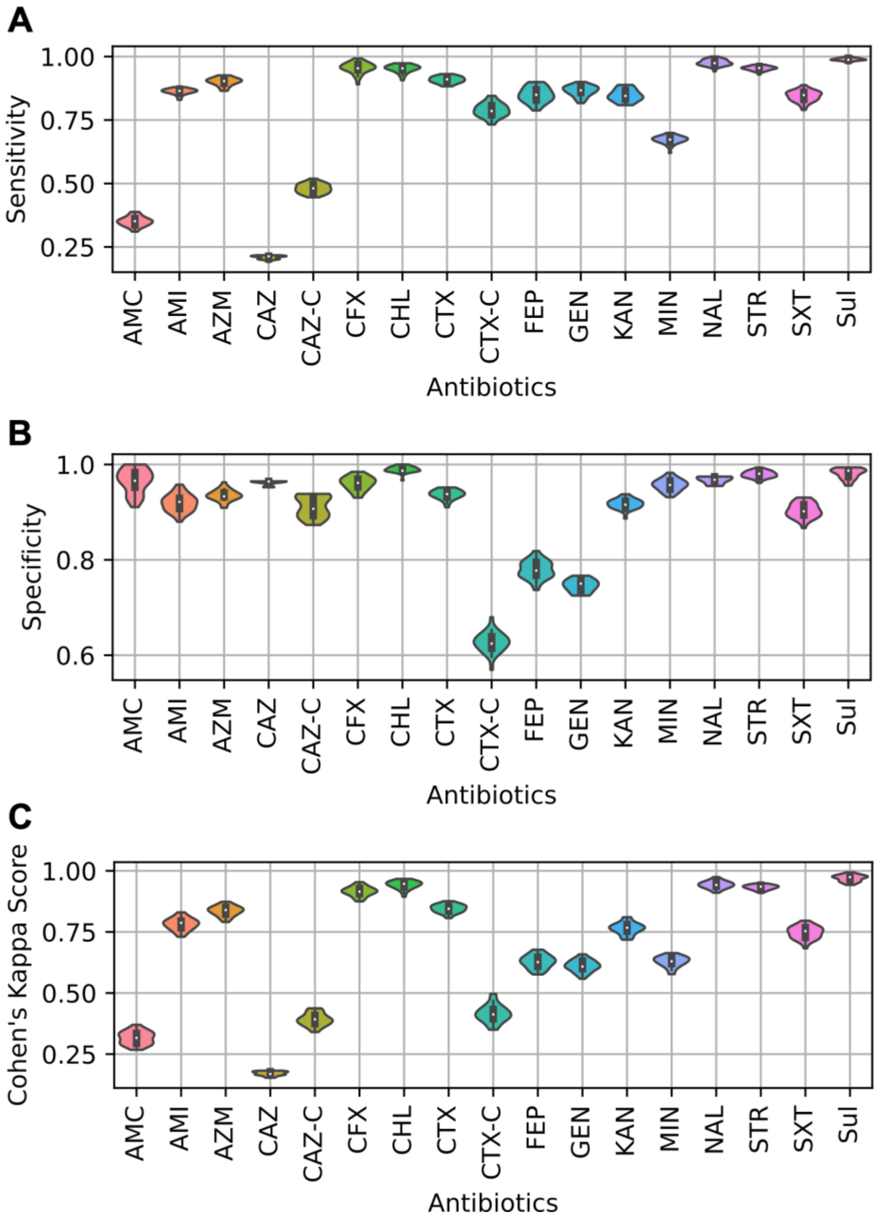

Supplementary Fig. 7. Machine learning performance from correlations between the gut microbiome and resistome, and antibiotic resistance in *E. coli*. Performance of the ML-powered predictive functions of *E. coli* resistance to specific antibiotics (ML technology: extra-tree classifier – see Materials and Methods). Performance indicators (A) sensitivity, (B) specificity and (C) Cohen's Kappa score were computed as the average of 30 iterations of nested cross-validation (see Materials and Methods). The violin plots show the distribution of the data, with each datapoint representing one antibiotic model. Inside each violin plot, a box plot is indicate with the box showing the interquartile range (IQR), the whiskers showing the rest of the distribution as a proportion of 1.5 x IQR, and the white circle showing the median value.

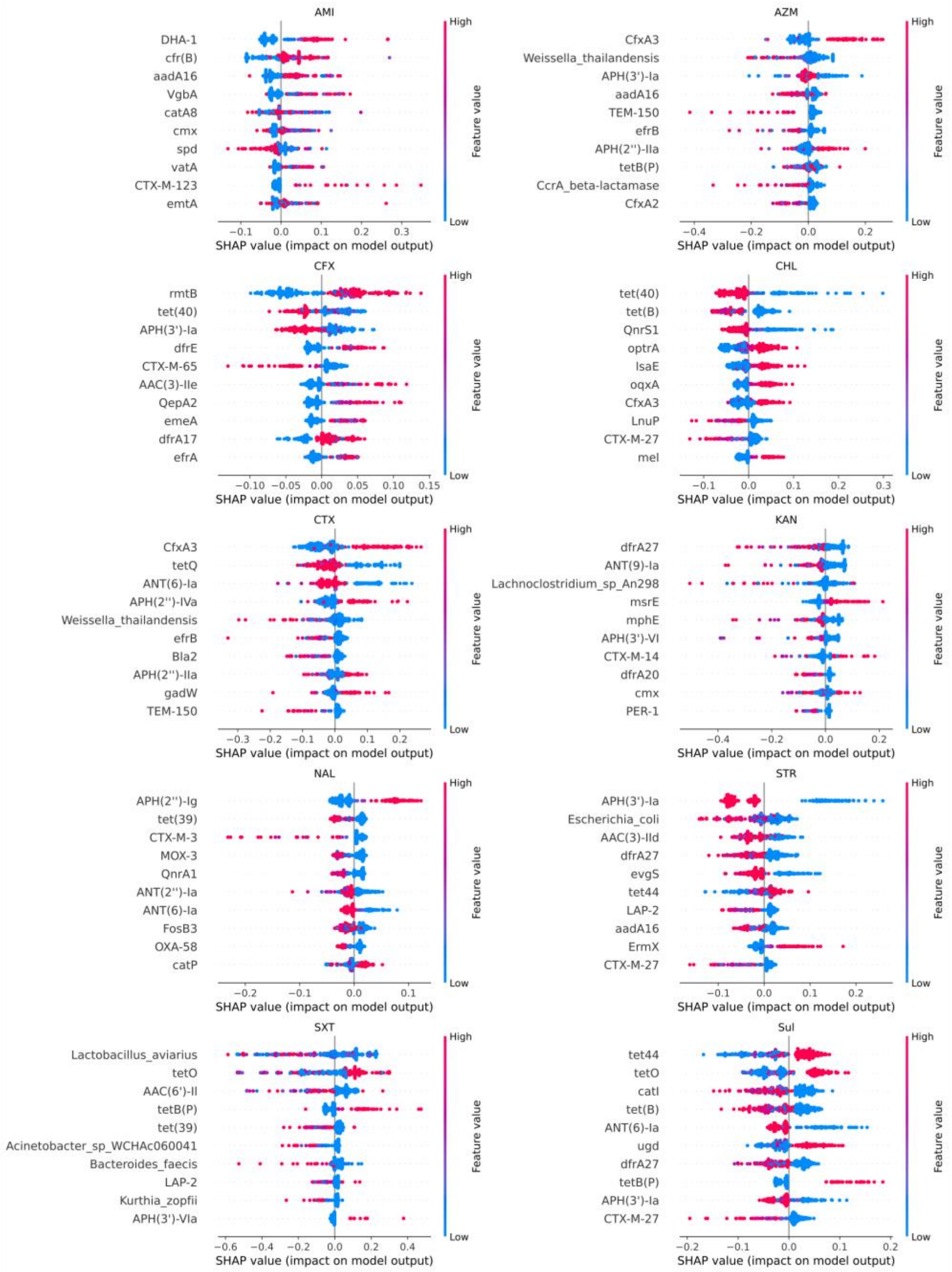

Supplementary Fig. 8. Bee swarm plot of SHAP-calculation for the ten highest ranking features for each of the antibiotic ML models with an AUC > 0.9. ARGs and microbial species are sorted by their mean absolute SHAP value in descending order with the most important features at the top. Each dot corresponds to one isolate in the study. The colour indicates either the ARG count or the microbial specie abundance normalized between 0 and 1. The bee swarm plot shows how the different feature in each isolate affects the prediction of the ML model towards resistance to the respective antibiotic.

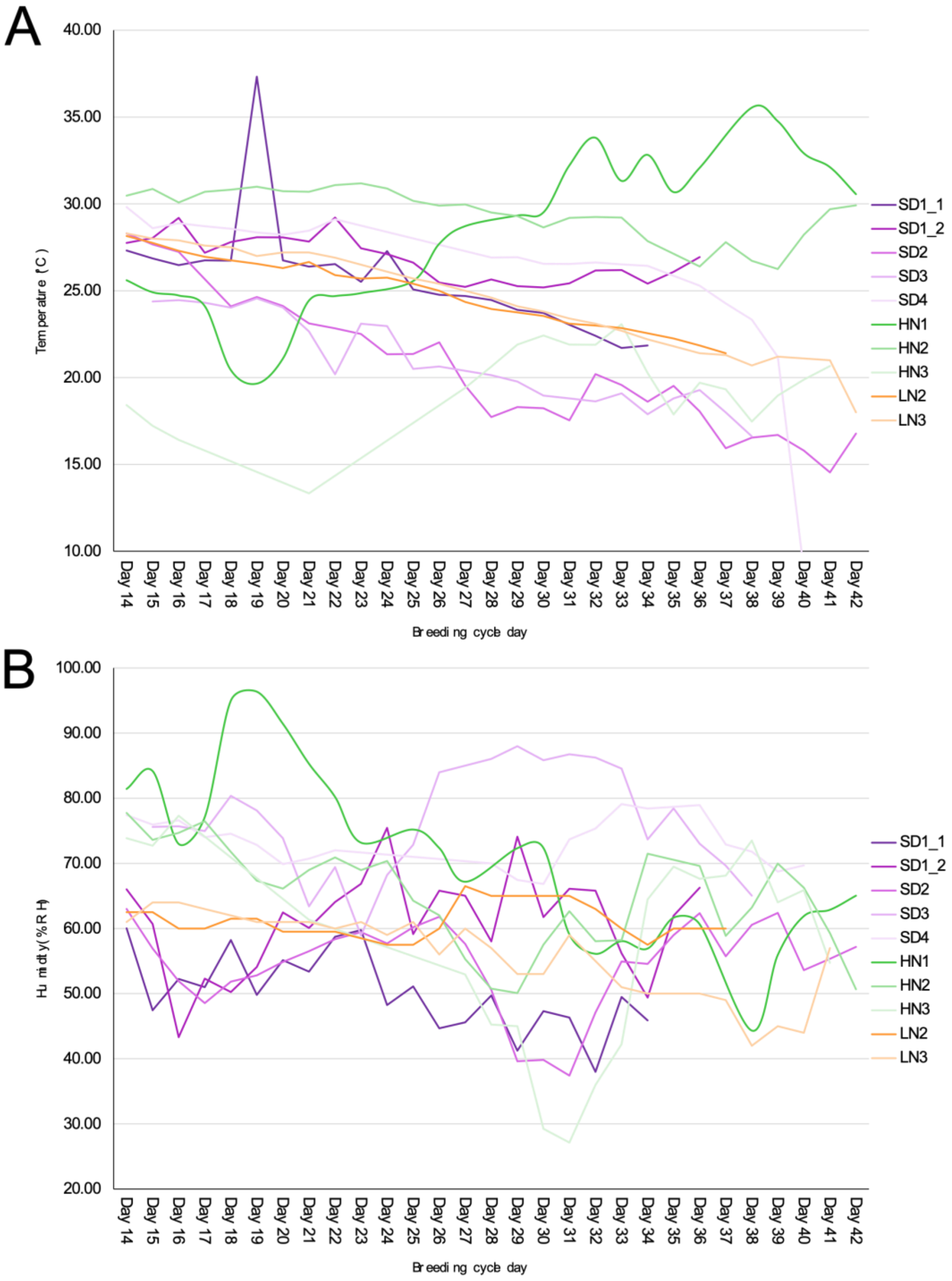

Supplementary Fig. 9. Daily average (A) Temperature and (b) Humidity measurements taken nine farms over the breeding cycles. LN1 is not shown as technical issues resulted in not data being collected.

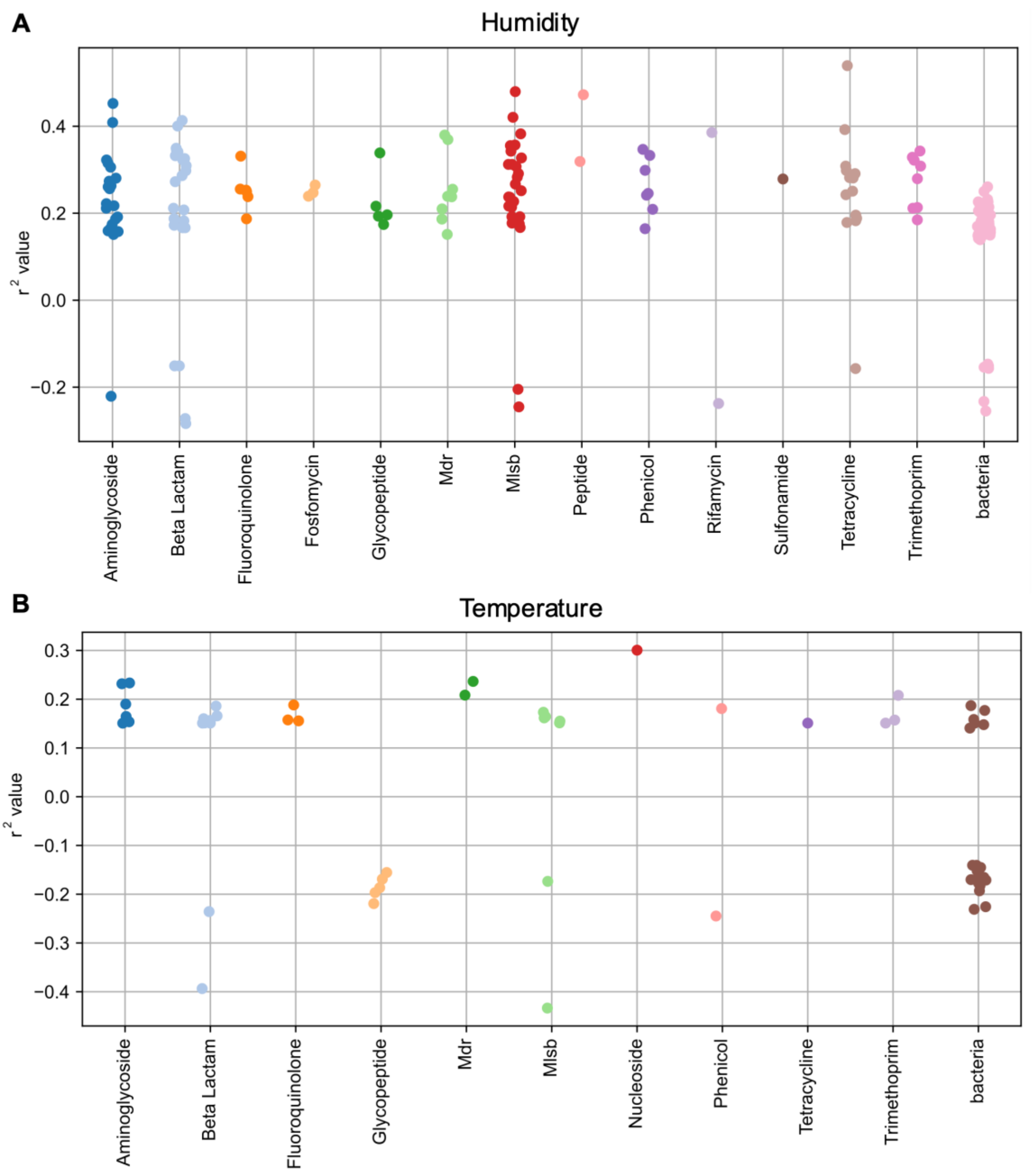

Supplementary Fig. 10. Categorical scatter plot of the statistically significant features selected by the regression analysis for the humidity and temperature. Each point on the plot represents a feature (ARG or microbial species) for which a regression analysis was performed. The R^2 value of the linear least-square regression analysis indicates the correlation between presence/absence of the ARGs and abundance of microbial species against temperature or humidity. The ARGs features are grouped by antibiotic class. R^2 ranges from -1 (negative correlation) to 1 (positive correlation).

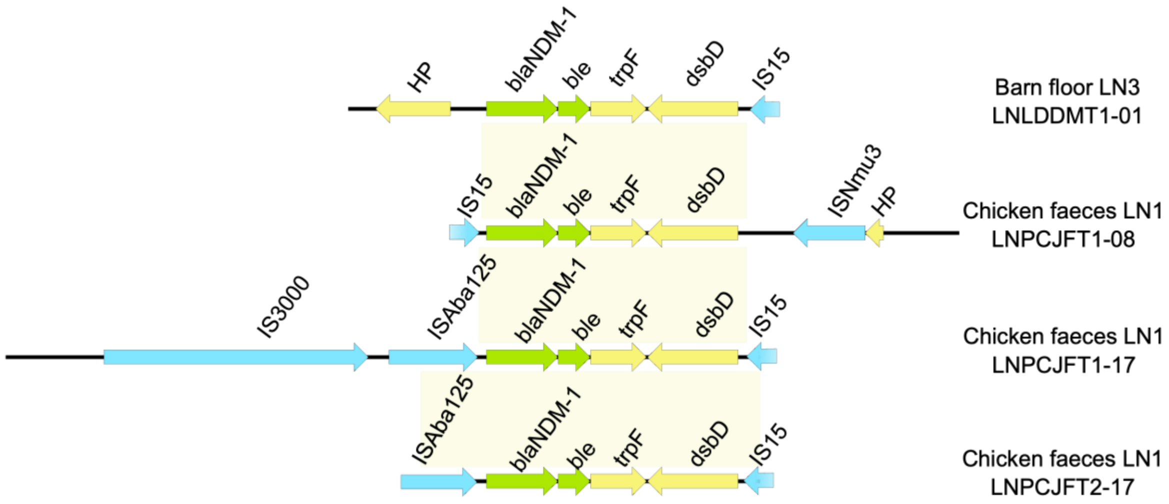

Supplementary Fig. 11. Gene structure of contigs carrying the mobile ARG pattern IS*15-NDM1* found in three chicken faeces samples and one barn floor sample. MGEs are represented by blue arrows, ARGs by green arrows and other genes by yellow arrows.

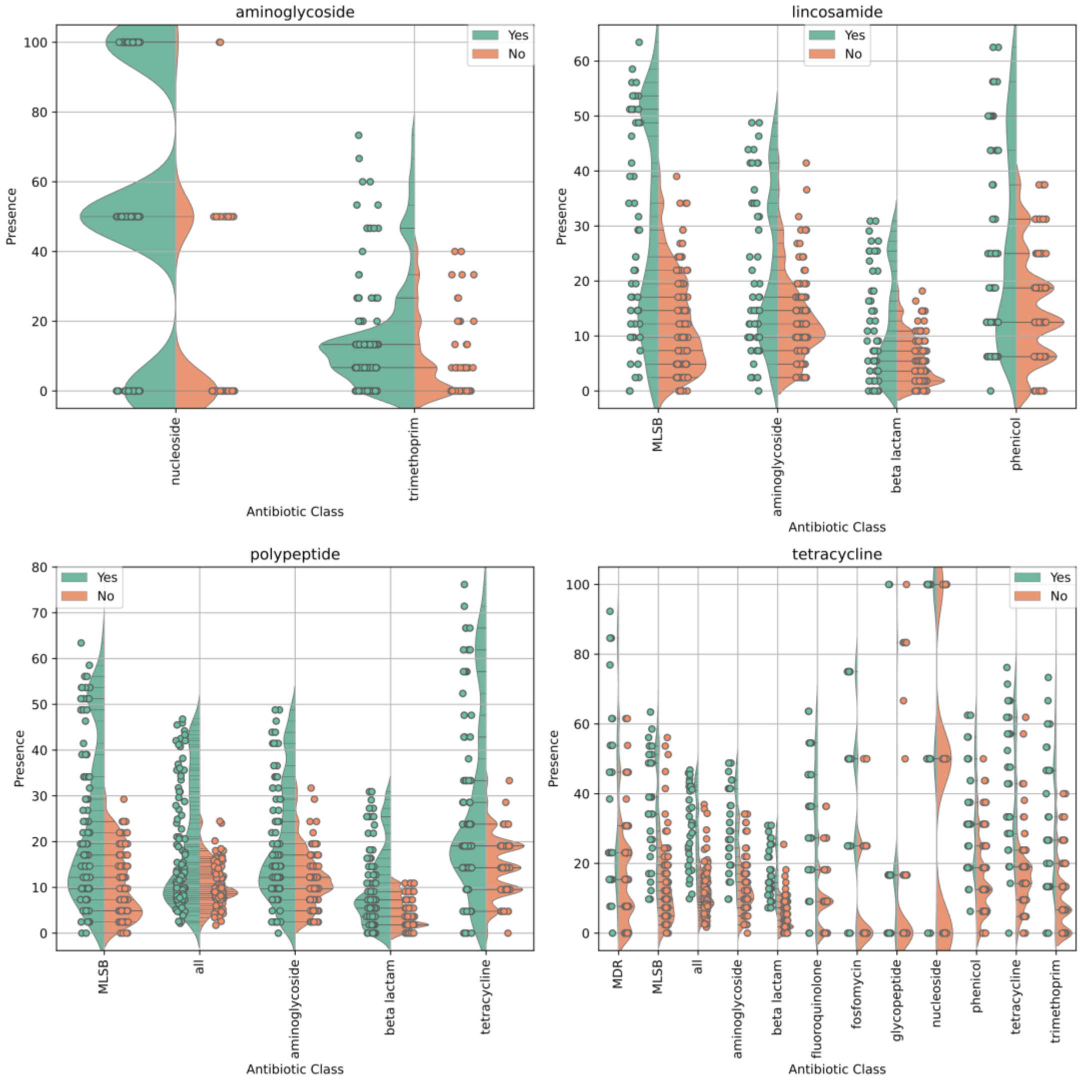

Supplementary Fig. 12. Antibiotic Usage analysis based on the ARGs' antibiotic class. Comparison of the ARGs' antibiotic classes, based on the ARGs selected by the machine learning framework (phase I and phase II, as described in the Materials and Methods) for the antibiotic models with an AUC > 0.9, across farms depending on antibiotic usage, i.e., the antibiotic was used or not. The antibiotic was considered present and labelled 'Yes' if it was used in the shed under study during the chicken production cycle, otherwise it was labelled 'No'. The y-axis refers to the percentage of ARGs from each class that were found in any individual sample relative to all the ARGs of that class found in the cohort. Only the statistically significant (two-sided Wilcoxon Rank-sum test with Holm Correction and adjusted p value < 0.05) ARGs' antibiotic classes are shown.

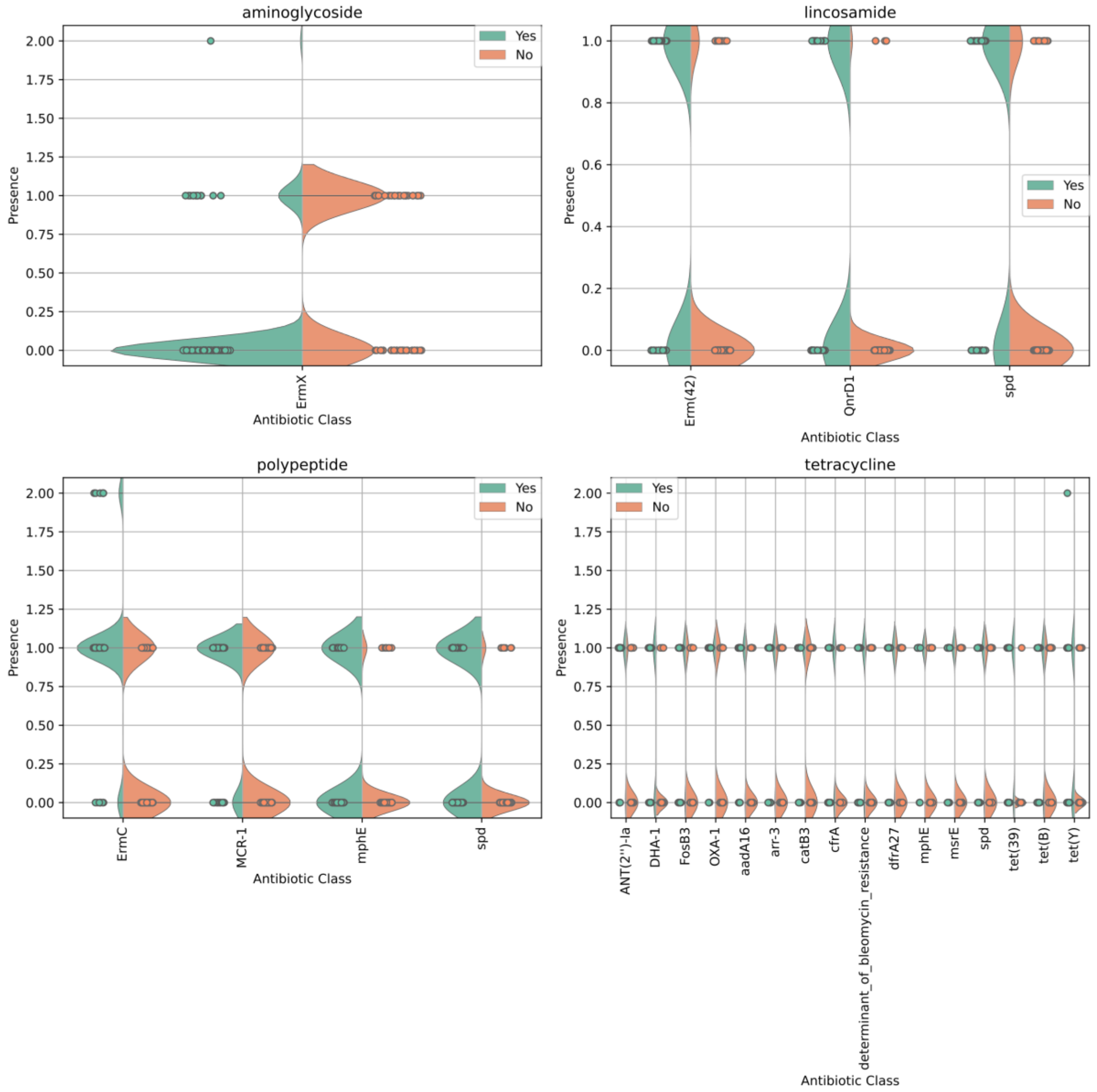

Supplementary Fig. 13. Antibiotic usage analysis based on count of ARGs. Comparison of the ARGs' count, selected by the machine learning framework, across the different farms depending on antibiotic usage, i.e. the antibiotic was used or not. Only the statistically significant (two-sided Wilcoxon Rank-sum test with Holm Correction and adjusted p value < 0.05) ARGs are shown.

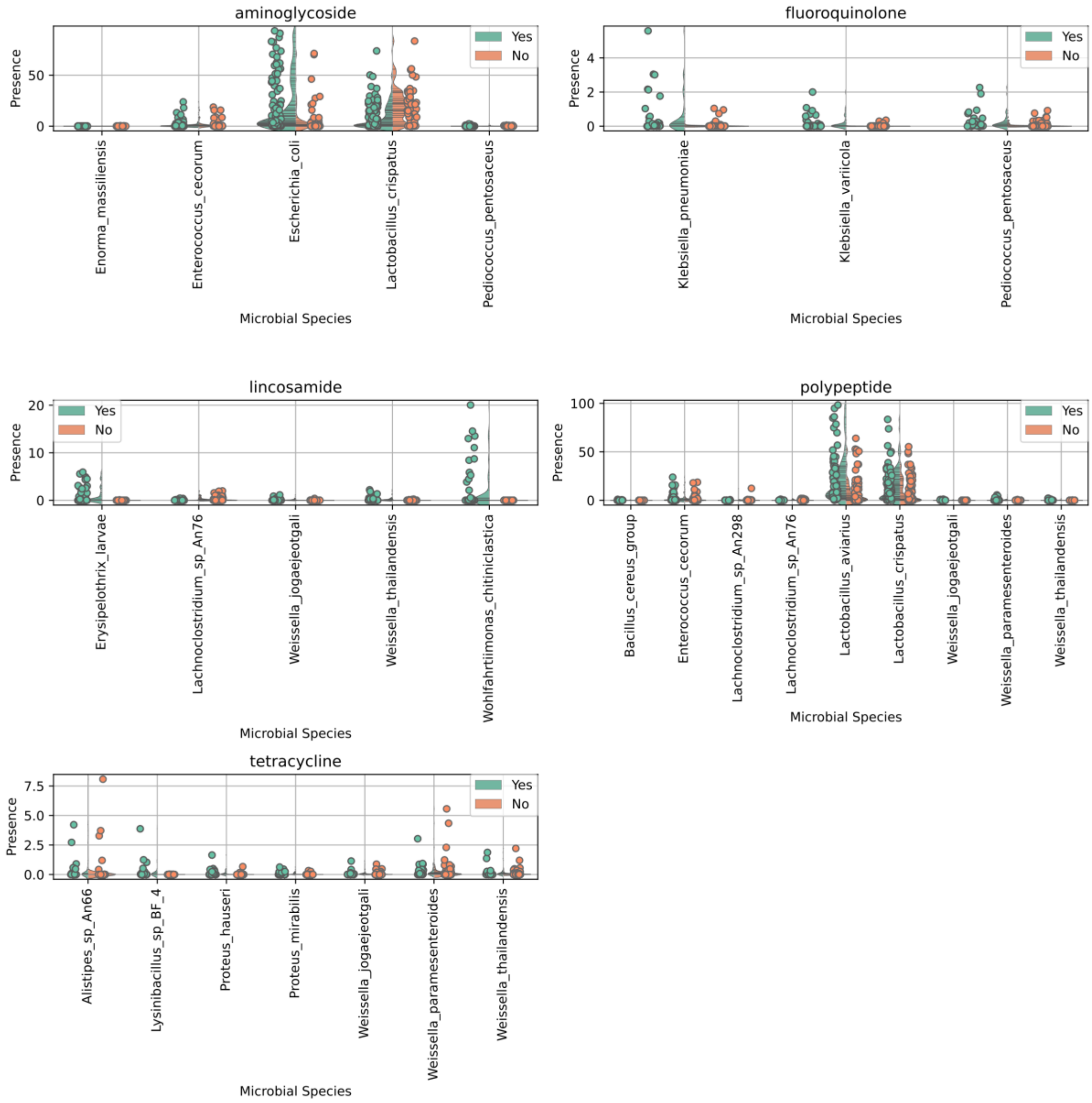

Supplementary Fig. 14. Antibiotic usage analysis based on relative abundance of microbial species. Comparison of microbial species abundances, selected by the machine learning framework, across the different farms depending on antibiotic usage, i.e. the antibiotic was used or not. Only the statistically significant (two-sided Wilcoxon Rank-sum test with Holm Correction and adjusted p value < 0.05) microbial species are shown.

List of Supplementary Tables

Supplementary Table 1. Sample Collection Information. This includes sample ID, sequence accession data, read counts, production cycle, sample collection date, sample source, sample collection place (Farm/Abattoir) and *E. coli* AMR testing results for the samples.

Supplementary Table 2. Summary of the number of samples collected at each farm separated by sample source.

Supplementary Table 3. Taxonomic profiling of the metagenomic samples based on MetaPhlAn 3.0 assignment.

Supplementary Table 4. Pairwise comparison of the mean relative abundance of phyla in different sample sources and farms, according to a two-sided Wilcoxon Rank-sum test. Holm correction applied to p values to account for multiple comparisons.

Supplementary Table 5. Pairwise comparison of the count of ARGs in different sample sources and farms, according to a two-sided Wilcoxon Rank-sum test. Holm correction applied to p values to account for multiple comparisons.

Supplementary Table 6. Shared mobile ARGs patterns of antimicrobial resistance genes (ARGs) and associated mobile genetic elements found within metagenomic samples. Contigs were classed as potentially mobile ARGs where the MGE was in close vicinity (5 kb) of the ARG. For each mobile ARG pattern analysed, the name and type of MGEs have been provided together with the sample and contig ID, source type, the names of closely related (>95% identity) variants, the distance between the ARG and MGE (in bp) and whether the ARG is clinically important.

Supplementary Table 7. Number of resistant and susceptible isolates included in this study

Supplementary Table 8. Prediction performance results of the predictive functions powered by machine learning for the correlation of the metagenome shotgun sequencing data (ARG presence-absence) and *E. coli* resistance/susceptibility profiles (isolates cultured from the same samples) against a panel of antimicrobials. The performance metrics were accuracy (TP+TN/(P+N)), sensitivity (true positive rate: TP/P), specificity (true negative rate: TN/N), AUC and precision. The scores for each performance metric were computed from 30 simulations using nested cross-validation. The mean ± standard deviation of these 30 iterations was then used as the result statistics for the performance.

Supplementary Table 9. Genes selected by the ML as being correlated with the AMR profiles of cultured *E. coli* isolates.

Supplementary Table 10. Daily average temperature and humidity data measured in the chicken housing over the breeding cycles at each of the ten farms.

Supplementary Table 11. Genes and microbial species significantly associated with temperature or humidity based on regression analysis using the temperature/humidity against each individual ARG count pattern and specie abundance. We report ARGs and species that obtained a slope significantly different from 0 using a two-sided t-test with a p-value of 0.05 and were present on the antibiotic models with an AUC > 0.9. For each ARG and specie we report the slope and interception values of the regression, the R2 value and the standard error. The R2 value will indicate how well the linear regression correlated with the points in our data, ranging from -1 to 1. Genes to test were selected based on them having a significant association with AMR profiles of cultured E. coli isolates.

Supplementary Table 12. Antibiotics administered to chickens in each farm over their lifetime following treatment protocols adopted by each farm.

Supplementary Table 13. Pairwise comparisons of microbial relative abundance and ARG count between samples taken from farms using an antibiotic and those not using an antibiotic.

Supplementary References

1 Forbes, J. D., Knox, N. C., Ronholm, J., Pagotto, F. & Reimer, A. Metagenomics: The Next Culture-Independent Game Changer. *Front. Microbiol.* **8**, doi:https://doi.org/10.3389/fmicb.2017.01069 (2017).

2 Maciel-Guerra, A. *et al.* Dissecting microbial communities and resistomes for interconnected humans, soil, and livestock. *The ISME Journal* **17**, 21-35, doi:https://doi.org/10.1038/s41396-022-01315-7 (2022).

3 Sun, J. *et al.* Environmental remodeling of human gut microbiota and antibiotic resistome in livestock farms. *Nature Commun* **11**, 1427, doi:https://doi.org/10.1038/s41467-020-15222-y (2020).

4 Katagiri, M. *et al.* Comprehensive genomic survey of antimicrobial-resistance bacteria in the sewage tank replacement with hospital relocation. *Infect Drug Resist* **14**, 5563-5574, doi:10.2147/IDR.S336418 (2021).

5 Kutilova, I. *et al.* Extended-spectrum beta-lactamase-producing *Escherichia coli* and antimicrobial resistance in municipal and hospital wastewaters in Czech Republic: Culture-based and metagenomic approaches. *Environ. Res.* **193**, 110487, doi:https://doi.org/10.1016/j.envres.2020.110487 (2021).

6 Ahmed, M. U. *et al.* Monitoring antimicrobial susceptibility of Neisseria gonorrhoeae isolated from Bangladesh during 1997-2006: emergence and pattern of drug-resistant isolates. *J. Health Popul. Nutr.* **28**, 443-449, doi:10.3329/jhpn.v28i5.6152 (2010).

7 Cheng, J. *et al.* Identification of pathogens in culture-negative infective endocarditis cases by metagenomic analysis. *Annals of Clinical Microbiology and Antimicrobials* **17**, 43, doi:https://doi.org/10.1186/s12941-018-0294-5 (2018).

8 Sanabria, A. M., Janice, J., Hjerde, E., Simonsen, G. S. & Hanssen, A.-M. Shotgun-metagenomics based prediction of antibiotic resistance and virulence determinants in *Staphylococcus aureus* from periprosthetic tissue on blood culture bottles. *Sci. Rep.* **11**, 20848, doi:10.1038/s41598-021-00383-7 (2021).

9 R. Hijmans & University of California Berkeley Museum of Vertebrate Zoology. *First-level Administrative Divisions, China, 2015*, <http://purl.stanford.edu/bw669kf8724> (2015). Last accessed 06 July 2023
