# Supplementary material for: Machine learning and metagenomics reveal shared antimicrobial resistance profiles across multiple chicken farms and abattoirs in China
Source: Nat Food. 2023 Aug 10;4(8):707–20. doi: 10.1038/s43016-023-00814-w (PMC10444626; doi:10.1038/s43016-023-00814-w)
Supplement: Supplementary file 2 — Reporting Summary [file 43016_2023_814_MOESM2_ESM.pdf]

nature portfolio

Corresponding author(s): Tania Dottorini

Last updated by author(s): Jul 5, 2023

Reporting Summary

Nature Portfolio wishes to improve the reproducibility of the work that we publish. This form provides structure for consistency and transparency in reporting. For further information on Nature Portfolio policies, see our Editorial Policies and the Editorial Policy Checklist.

Statistics

For all statistical analyses, confirm that the following items are present in the figure legend, table legend, main text, or Methods section.

n/a	Confirmed	
☐	☒	The exact sample size (n) for each experimental group/condition, given as a discrete number and unit of measurement
☐	☒	A statement on whether measurements were taken from distinct samples or whether the same sample was measured repeatedly
☐	☒	The statistical test(s) used AND whether they are one- or two-sided<br>*Only common tests should be described solely by name; describe more complex techniques in the Methods section.*
☒	☐	A description of all covariates tested
☐	☒	A description of any assumptions or corrections, such as tests of normality and adjustment for multiple comparisons
☐	☒	A full description of the statistical parameters including central tendency (e.g. means) or other basic estimates (e.g. regression coefficient) AND variation (e.g. standard deviation) or associated estimates of uncertainty (e.g. confidence intervals)
☐	☒	For null hypothesis testing, the test statistic (e.g. F, t, r) with confidence intervals, effect sizes, degrees of freedom and P value noted<br>*Give P values as exact values whenever suitable.*
☐	☒	For Bayesian analysis, information on the choice of priors and Markov chain Monte Carlo settings
☒	☐	For hierarchical and complex designs, identification of the appropriate level for tests and full reporting of outcomes
☒	☐	Estimates of effect sizes (e.g. Cohen's d, Pearson's r), indicating how they were calculated

Our web collection on statistics for biologists contains articles on many of the points above.

Software and code

Policy information about availability of computer code

Data collection	Sensor data collected through vendor-proprietary data loggers, available in xls or csv formats

Data analysis

Our code is available on Github: https://github.com/tan0101/Commercial_MGS2023
For the bioinformatics analysis the following software were used:
Readfq (V8, https://github.com/cjfields/readfq)
Bowtie2 v2.3.4.1
SAMtools v1.9
MEGAHIT software v1.1.2
BWA MEM v2-2.1
METABAT2v2.15
MetaPhlAn v3.0
Rv3.6.2
To perform the ML and data analysis anaylsis the following software were used:
IQ-tree2v2.0.6
BEASTv1.10.4
Tracer v1.7.1
python (v3.9.15)
scikit-learn(v1.0.2),
scipy (v1.9.3)
networkx (v2.8.4)

For manuscripts utilizing custom algorithms or software that are central to the research but not yet described in published literature, software must be made available to editors and reviewers. We strongly encourage code deposition in a community repository (e.g. GitHub). See the Nature Portfolio guidelines for submitting code & software for further information.

Data

Policy information about availability of data

All manuscripts must include a data availability statement. This statement should provide the following information, where applicable:

- Accession codes, unique identifiers, or web links for publicly available datasets
- A description of any restrictions on data availability
- For clinical datasets or third party data, please ensure that the statement adheres to our policy

The metagenomic sequencing data supporting the conclusions of this article are available in the NCBI database under Bioproject accession numbers PRJNA678871 (for Shandong 1_1 and 1_2) and PRJNA841806 (for all other farms) available on: https://www.ncbi.nlm.nih.gov/bioproject/PRJNA678871 and https://www.ncbi.nlm.nih.gov/bioproject/PRJNA841806. In addition the reference genome used for filtering host DNA is available in NBCI database under accession GCF_000002315.6 https://www.ncbi.nlm.nih.gov/datasets/genome/GCF_000002315.6/.

Research involving human participants, their data, or biological material

Policy information about studies with human participants or human data. See also policy information about sex, gender (identity/presentation), and sexual orientation and race, ethnicity and racism.

Reporting on sex and gender	n/a
Reporting on race, ethnicity, or other socially relevant groupings	n/a
Population characteristics	n/a
Recruitment	n/a
Ethics oversight	n/a

Note that full information on the approval of the study protocol must also be provided in the manuscript.

Field-specific reporting

Please select the one below that is the best fit for your research. If you are not sure, read the appropriate sections before making your selection.

☒ Life sciences ☐ Behavioural & social sciences ☐ Ecological, evolutionary & environmental sciences

For a reference copy of the document with all sections, see nature.com/documents/nr-reporting-summary-flat.pdf

Life sciences study design

All studies must disclose on these points even when the disclosure is negative.

Sample size

For the analysis of how microbial communities and resistomes are differentiated across farm sources and between farm and abattoir, we did not perform sample size calculation as this was an observational/exploratory study.
For the results demonstrating the proposed approach to the AMR surveillance (based on ML prediction of resistant phenotypes), sample size

	is based on achieving desired power in the predictor. For binary classifiers, power is the sensitivity (true positive rate, defined as 1-beta, where beta is the false negative rate, i.e. type II error (Banerjee, A. et al. 2009, Industrial psychiatry journal). Note that the type II error is particularly relevant for resistance, as it implies a resistant phenotype escaping detection (Mahfouz, N. et al. 2020, Journal of Antimicrobial Chemotherapy). Using 191 samples, we achieved an average power of 92% for the 11 antibiotic models studied. We also wanted to identify the minimum number of samples required to achieve at least 80% sensitivity (power). Because for classifiers based on ML (e.g. SVMs, decision trees, random forest, adaboost, neural networks), sample size calculation to achieve power is not directly possible using conventional analytical methods (Li. J. et al. 2020, Patterns), we applied a bespoke iterative method (wrapper backward selection - WBS, Figueroa, R et al. 2012, BMC Medical Informatics and Decision Making) as done in our previous paper (Maciel-Guerra, A. et al. 2022, The ISME Journal). The method estimates how power decreases with smaller sample sizes. In our case, WBS estimated the need of 160 samples on average, to achieve 80% power, which is less to what we used (191).
Data exclusions	No data were excluded.
Replication	At least three biological replicates per sample were taken, all were successful.
Randomization	Biological samples were collected randomly without knowing the AMR phenotypes.<br>For the analysis of how microbial communities and resistomes are differentiated across farm sources and between farm and abattoir, random assignment to groups was not performed as this is an exploratory/observational study.<br>For the ML classification the samples were randomly assigned to training and testing groups using a nested cross validation procedure (30 iterations per classifier).
Blinding	Biological samples were collected randomly without knowing the AMR phenotypes.

Reporting for specific materials, systems and methods

We require information from authors about some types of materials, experimental systems and methods used in many studies. Here, indicate whether each material, system or method listed is relevant to your study. If you are not sure if a list item applies to your research, read the appropriate section before selecting a response.

Materials & experimental systems

n/a	Involved in the study	
☒	☐	Antibodies
☒	☐	Eukaryotic cell lines
☒	☐	Palaeontology and archaeology
☒	☐	Animals and other organisms
☒	☐	Clinical data
☒	☐	Dual use research of concern
☒	☐	Plants

Methods

n/a	Involved in the study	
☒	☐	ChIP-seq
☒	☐	Flow cytometry
☒	☐	MRI-based neuroimaging
